# N-2 Repetition Costs in Task Switching: Task Inhibition or Interference Between Task Episodes?

**DOI:** 10.5334/joc.244

**Published:** 2022-11-04

**Authors:** Stefanie Schuch, Emily Keppler

**Affiliations:** 1 Institute of Psychology, RWTH Aachen University, Germany

**Keywords:** task switching, N-2 task repetition costs, episodic interference, task inhibition

## Abstract

A prominent behavioral marker of inhibition in task switching is the “N-2 repetition cost” that denotes the decrement in performance in task sequences with an N-2 task repetition (ABA), relative to task sequences without an N-2 task repetition (CBA). Recently, it has been critized that N-2 repetition costs at least partially reflect interference between task episodes, rather than persisting inhibition, raising doubts about the interpretation of N-2 repetition costs as a measure of inhibition. Here, we aim to generalize these conclusions in two ways. First, we define episodic effects in task switching with respect to the last episode of the same task, which might have occurred several trials back (e.g., in trial N-2, N-3, etc.). Second, we distinguish between episodic interference caused by task-relevant and task-irrelevant features. We present a re-analysis of previously published data, and a new pre-registered experiment, where we manipulated the degree of interference between task episodes in three levels (episodic match of both task-relevant and task-irrelevant features, episodic match of only task-relevant features, episodic mismatch of both kinds of features). We observed empirical evidence for both cognitive mechansims: Episodic interference was indicated by a main effect of episodic condition; task-level inhibition was indicated by N-2 repetition costs, and by a performance benefit with increasing task lag in an exploratory task-lag analysis. We did not observe any significant modulation of N-2 repetition costs by episodic condition, suggesting that if there was such a modulation, this effect appears to be smaller than the individual contributions of episodic interference and inhibition to task performance.

## Introduction

Task inhibition is considered a cognitive control process that facilitates switching from one cognitive task to another. The no-longer relevant mental representation of a cognitive task (task-set) becomes inhibited in order to reduce interference with the mental representation of the new task. A prominent behavioral measure of such task-level inhibition is the “N-2 task repetition cost” (also termed “backward inhibition effect”; Mayr & Keele, 2000; see Koch et al., 2010, for review) that denotes the decrement in performance in task sequences with an N-2 task repetition (sequences of type ABA), relative to task sequences without an N-2 task repetition (sequences of type CBA). The reasoning is that during the switch from task A to task B, task A becomes inhibited, and this inhibition decays slowly over time. Therefore, the sooner one switches back to the previously inhibited task A, the more persisting inhibition needs to be overcome. Hence, performance is worse in ABA (switching back to task A after just one intermediate trial) than CBA sequences (switching back to task A after at least two intermediate trials). This measure of task-level inhibition has been applied in various clinical applications, in order to assess whether cognitive inhibition is altered in psychiatric or neurological conditions, such as in depressive rumination (De Lissnyder et al., 2010; Whitmer & Banich, 2007, 2012; Whitmer & Gotlib, 2012), obsessive-compulsive disorder (Moritz et al., 2004), Parkinson’s disease (Fales et al., 2006), or frontal lobe damage (Mayr et al., 2006). It has also been applied to investigate cognitive inhibition in healthy populations, assessing differences in inhibitory functions between individuals (Pettigrew & Martin, 2016; Rey-Mermet et al., 2018), between different age groups (e.g., Lawo et al., 2012; Schuch, 2016; Schuch & Konrad, 2017), and between bilinguals and monolinguals (Prior, 2012).

**Figure 1:**
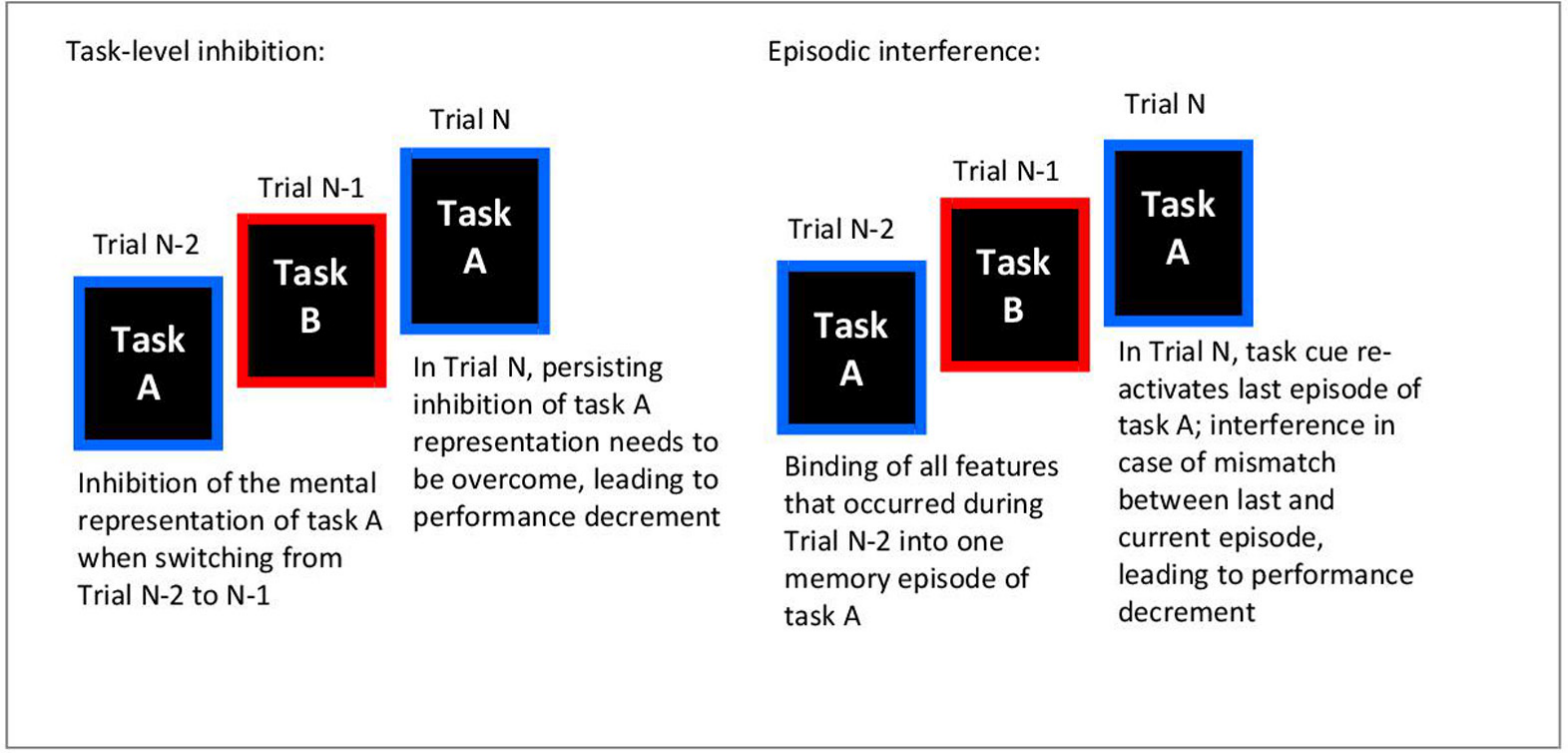
Schematic illustration of the two different cognitive mechanisms (task-level inhibition and episodic interference) that could be contributing to N-2 task repetition costs.

Given this wide range of applications, it is important to ensure that N-2 task-repetition costs are a valid measure of inhibitory control on the task level. There is some recent debate, however, as to whether N-2 task-repetition costs are a good empirical measure of task-level inhibition. An alternative explanation for N-2 task repetition costs is that they (at least partly) reflect interference between different task episodes (– an issue first raised by Mayr, 2002, and further investigated by Gade et al., 2016; Grange, 2018; Grange et al. 2017, 2019; Kowalczyk & Grange, 2019). According to this alternative explanation, the task cue in trial N re-activates the mental representation (memory trace) of the last episode of this task (including all features of that episode, such as task cue, set of relevant response rules, stimulus, response). In case of a mismatch between previous and current episode (e.g., a different stimulus and response), there is interference between the episodes, leading to a decrement in performance when processing the current episode. The task inhibition account and the episodic interference account of N-2 repetition costs describe two different cognitive mechanisms (see Figure 1 for a graphical illustration): Whereas the inhibition account presumes that the performance decrement measured in trial N reflects an aftereffect of a previously applied control process (i.e., inhibition of the N-2 task representation, which had occurred during the switch from trial N-2 to trial N-1), the episodic interference account assumes that the performance decrement is due to cognitive processes triggered during trial N processing: The onset of the trial N task cue is thought to trigger re-activation (or “retrieval”) of the most recent episode of this task, which leads to interference with processing of the current task episode.

### Episodic interference accounts in cognitive psychology

Episodic-binding-and-retrieval accounts are widely discussed in cognitive psychology as a viable alternative explanation for a range of empirical effects that have previously been explained by several different, isolated, theoretical accounts. Generally speaking, episodic interference accounts assume that features that occur together during one moment in time (e.g., the stimulus and the response that occur during one trial in an experiment) are integrated (or “bound together”) into an episodic memory representation. If then, at a later point in time, one of the features is activated again (e.g., when the same stimulus is presented again in a later trial), this triggers re-activation (or “retrieval”) of the complete memory episode. Such retrieval may facilitate or impair performance depending on whether the retrieved episode and the current episode match or do not match. Episodic binding accounts have been applied to account for performance in single-task paradigms, explaining effects of stimulus repetition or response repetition from one trial to the next (e.g., Davelaar & Stevens, 2009; Hommel et al., 2004; Mayr et al., 2003), or negative priming effects where a previously to-be-ignored distractor stimulus becomes the target stimulus in the subsequent episode (e.g., Neill, 1997). Comprehensive models of episodic binding have been developed that consider a wide range of episodic features beyond stimulus and response, such as action plan, task context, internal control states, post-response action effects, and affective connotations (e.g., Frings et al., 2020; Hommel, 2004, 2019; Hommel et al. 2001; Schmidt et al., 2016, 2020; see also Abrahamse et al., 2016; Dignath et al., 2019; Egner, 2014; Foerster et al., 2022; Spapé & Hommel, 2008, Whitehead et al., 2020, for binding of cognitive control states into episodic memory representations).

Even within modern binding-and-retrieval frameworks, several open questions remain (see, e.g., Frings et al., 2020). For instance, regarding the formation of an episode, are all features given the same weight? Or is there a different weighting of the episodic features within an episode, such that task-relevant features, or more salient features, are being given more weight than task-irrelevant features? (e.g., Frings & Rothermund, 2011; Memelink & Hommel, 2013; Qiu et al., 2022). Regarding the retrieval of episodes, open question are: Is only the most recent episode retrieved, or are all previous episodes that are associated with a certain feature retrieved? If all are retrieved, is there a different weighting of the episodes, e.g., according to their recency? Relatedly, do episodic memory representations decay over time, and if so, how quickly? While some models explicitly assume temporal decay of the episodic representations (e.g., Schmidt et al., 2016), and there is empirical evidence for decay of episodic bindings over time (Hommel & Frings, 2020), there is also evidence that episodic bindings can “survive” several intervening trials, and can influence performance in trials that occur much later in the experiment (e.g., Moutsopoulou et al., 2015, 2019; Waszak et al., 2003).

Another open question refers to how exactly to define the degree of episodic match between episodes. One possibility would be to count the number of overlapping features between the episodes; the more features repeat, the larger the episodic match, and the greater the episodic facilitation effect. Another possibility would be to also consider the number of non-overlapping features between the episodes; the more features differ between the episodes, the greater the episodic interference; the net episodic effect would then be an aggregate of the episodic facilitation and interference components (e.g., Altmann, 2011). If no features at all repeat between two episodes, the earlier episode is not retrieved at all when processing the later episode, such that neither episodic facilitation nor interference occurs. In line with this notion, it has been observed in single-task context that when no features at all repeat between two subsequent trial episodes, performance is better than when one feature repeats but the other features do not (“partial repetition costs”,^1^ e.g., Hommel, 2004). In task-switching paradigms, on the other hand, such partial repetition costs are not always observed; for instance, performance in task switches has been found to be unaffected by whether an irrelevant feature of the task cue repeats or switches (e.g., Benini et al., 2022; Kandalowski et al., 2020; Koch, et al., 2018).

Furthermore, in task switching paradigms, the situation is more complex than in single-task paradigms, because usually two stimuli are presented in each trial: the task cue, and the task-relevant stimulus that needs to be acted upon. Both of these stimuli probably re-activate the episode they have last been bound to, such that there can be two different memory episodes that interfere with processing of the current task episode. One possibility to get around this problem is to make the simplifying assumption that interference between the higher-level task-set representations plays a larger role (and influences performance to a larger degree) than interference between lower-level features such as the target stimulus presented within an episode. Under this assumption, it would be most important to consider the most recent episode of the same task (which might have occurred in trial N-1, N-2, or further back) for determining episodic interference effects in a task-switching paradigm. Another possibility is to make the simplifying assumption that the most recent (i.e., trial N-1) trial episode interferes most with the current trial episode, and hence to only consider episodic interference effects with respect to the immediately preceding trial (i.e., N-1), while neglecting all trials further back (i.e., N-2, N-3, etc.). The latter approach was adopted, for instance, by Altmann (2011), who presented a formal model of episodic binding and retrieval effects in task switching, with an emphasis on response-repetition effects. Focusing on the relationship between trials N and N-1, Altmann (2011) proposed that whenever a feature of the immediately preceding task episode is repeated, this re-activates the complete previous trial N-1 episode, and the more features of the previous episode repeat, the greater the episodic facilitation effect.^2^

Which features of an episode need to be distinguished in a task-switching paradigm? Altmann (2011) suggested to consider four different features of a task episode: task cue, task set, stimulus, and response. Depending on the paradigm, however, the list of features may differ; for instance, in some task-switching experiments, there is no task cue, but the task sequence needs to be retrieved from memory. Moreover, it might be important to further distinguish between stimulus identity as one feature (e.g., the digit “7”), and stimulus category as another feature (e.g., the stimulus category “odd”) when categorization tasks are used in the task-switching paradigm (e.g., tasks such as “categorize stimulus parity; for odd digits, press left button; for even digit, press right button”). Furthermore, it might be important to distinguish between the physical response (e.g., left button press) and the task-specific semantic meaning of the response (e.g., “odd number”) as different features (see, e.g., Giesen & Rothermund, 2016). In some accounts, the applied S-R binding (e.g., “if number is odd, press left key”) is considered a feature of a task episode (e.g., Frings et al., 2020), which is sometimes termed “response rule” or “action rule”. Hence, the question of which, and how many, features need to be considered turns out to be a nontrivial issue.

### Disentangling inhibition and episodic interference in N-2 task-repetition costs

**The “N-2 contrast”.** Returning to N-2 task repetition costs in task switching, how can the contributions of inhibition and episodic interference to this measure be disentangled empirically? Mayr (2002) suggested a methodology that has since been adopted in several research papers (e.g., Grange, 2018; Grange et al., 2017; 2019; Kowalczyk & Grange, 2019). In particular, Mayr (2002) developed a task-switching paradigm where participants switched between three different tasks, which were three different spatial transformation rules. In each trial, they were presented with a visual stimulus in one of the four corners of a square, and they had to press one out of four response keys that were also arranged in a square-like fashion. A task cue presented at the beginning of each trial indicated which of three different spatial transformation rules (i.e., tasks) to apply: Transform vertically (e.g., an upper left stimulus would require a lower left response), transform horizontally (e.g., an upper left stimulus would require an upper right response), or transform diagonally (e.g., an upper left stimulus would require a lower right response).

In this paradigm, Mayr (2002) compared N-2 task repetition costs in two conditions: N-2 response repetitions and N-2 response switches. We term this the “N-2 contrast” of N-2 task repetition costs. The reasoning was the following: In an ABA task sequence, the task cue presented in trial N will re-activate the previous episode of this task (which occurred in trial N-2). If stimulus and response match between N-2 and N, this should lead to episodic facilitation; if they do not match, this should lead to episodic interference. Hence, one would expect better performance in ABA task sequences with N-2 response repetitions than in ABA task sequences with N-2 response switches. In CBA task sequences, the task cue presented in trial N will not re-activate the N-2 task episode (as it was a different task); therefore, it should not make a difference whether the responses in N-2 and N did or did not match, and performance should not differ between CBA task sequences with N-2 response repetition or N-2 response switch.

Mayr (2002) observed N-2 task repetition costs that did not differ statistically between the two response-transition conditions (i.e., the interaction between the factors N-2 Task Transition and N-2 Response Transition was not significant), and concluded that the N-2 task-repetition costs were due to task-level inhibition, and were not modulated by episodic interference. However, as noted by Grange et al. (2017), the lack of an interaction could be due to a lack of statistical power for detecting the interaction; numerically, performance in ABA sequences with N-2 response switches was worse than in ABA sequences with N-2 response repetitions in Mayr’s data, which could point to an influence of episodic retrieval. Indeed, Grange et al. (2017) replicated and extended the work of Mayr (2002) using the same task-switching paradigm, but with more statistical power, and observed that N-2 task-repetition costs were smaller in N-2 response repetitions than in N-2 response switches (i.e., the interaction between the two factors was significant), suggesting that episodic interference did contribute to the observed N-2 task repetition cost (see also Grange, 2018; Grange et al., 2019; Kowalczyk & Grange, 2019).

Critically, however, in both Mayr (2002) and Grange et al. (2017), N-2 task repetition costs were still observed in the condition with N-2 response repetitions, suggesting that task-level inhibition was still present. If task-level inhibition did not exist, one would expect an N-2 task repetition *benefit* in this condition due to episodic facilitation, which is not what was observed. Grange et al. (2017) concluded that episodic interference contributes to the empirically observed N-2 task repetition costs, but cannot fully account for it. Hence, both persisting task inhibition and episodic interference seem to contribute to N-2 task repetition costs. Grange et al. (2017) suggested to take the reduced N-2 task repetition costs in N-2 response repetitions as a “purer” measure of task-level inhibition that controls for episodic retrieval effects (see also Grange, 2018; Grange et al., 2019; Kowalczyk & Grange, 2019). However, N-2 task repetition costs in N-2 response repetitions likely underestimate the true inhibition effect, because inhibition and episodic retrieval effects work in opposing directions in this condition.

**Potential shortcomings of the “N-2 contrast”.** Assessing the contribution of episodic retrieval with the “N-2 contrast” method developed by Mayr (2002) is a promising first approach. However, a potential shortcoming of Mayr’s “N-2 contrast” is that episodic interference effects from task episodes earlier than trial N-2 are not considered. In experimental task-switching paradigms, when switching to a task, this task has likely been previously performed even in CBA task sequences (in trial N-3 or further back). It is thus possible that performance in the CBA conditions is also influenced by episodic interference effects. Specifically, the task cue for the current task A might re-activate the most recent task A episode (which was performed in trial N-3 or further back), and performance might depend on the degree of episodic match between the current and previous episode of this task. It is therefore important to control for episodic interference effects not only in ABA, but also in CBA task sequences. For instance, if more episodic mismatches occurred in CBA than ABA task sequences (or vice versa), this would systematically bias the estimation of N-2 task repetition costs.

As discussed above, several theories assume that there is temporal decay of memory episodes (e.g., Schmidt et al., 2016; see Frings et al., 2020 for an overview). In this case, the episodic interference effects observed in CBA task sequences should be smaller than those in ABA task sequences, because more time has passed since the last execution of task A in CBA than in ABA sequences. Importantly though, episodic interference effects could still play a role in CBA task sequences. The strength of such longer-lag episodic interference effects in the CBA condition would depend on the lag between present and previous task episode, and would become smaller with increasing lag.

Apart from the consideration of longer-lag episodic interference effects, another issue with Mayr’s “N-2 contrast” is the assumption that performance does not differ between N-2 response repetitions and N-2 response switches in CBA task sequences. As outlined above, the reasoning underlying the “N-2 contrast” is that in CBA task sequences, the task cue presented in trial N will not re-activate the N-2 task episode (as it was a different task); therefore, there should be no episodic interference between the task episodes of trial N and trial N-2, and it should not make a difference whether the responses in trial N and trial N-2 did or did not match. In the original study by Mayr (2002), this assumption was met: Performance in the two CBA conditions (CBA with N-2 response repetitions versus CBA with N-2 response switches) was indeed very similar in both RTs and error rates. In the subsequent work by Grange and colleagues, this assumption was often but not always met. In some of the studies, there were performance differences between the two CBA conditions, with worse performance in N-2 response repetitions than N-2 response switches.^3^ This difference between CBA conditions was numerically in the same order of magnitude as the ABA-CBA difference in those studies (although it was not analyzed statistically). If such a difference between the CBA conditions is observed, however, the results are difficult to interpret, because neither the inhibition account nor the episodic retrieval account would predict such a difference between the CBA conditions.

**Figure 2:**
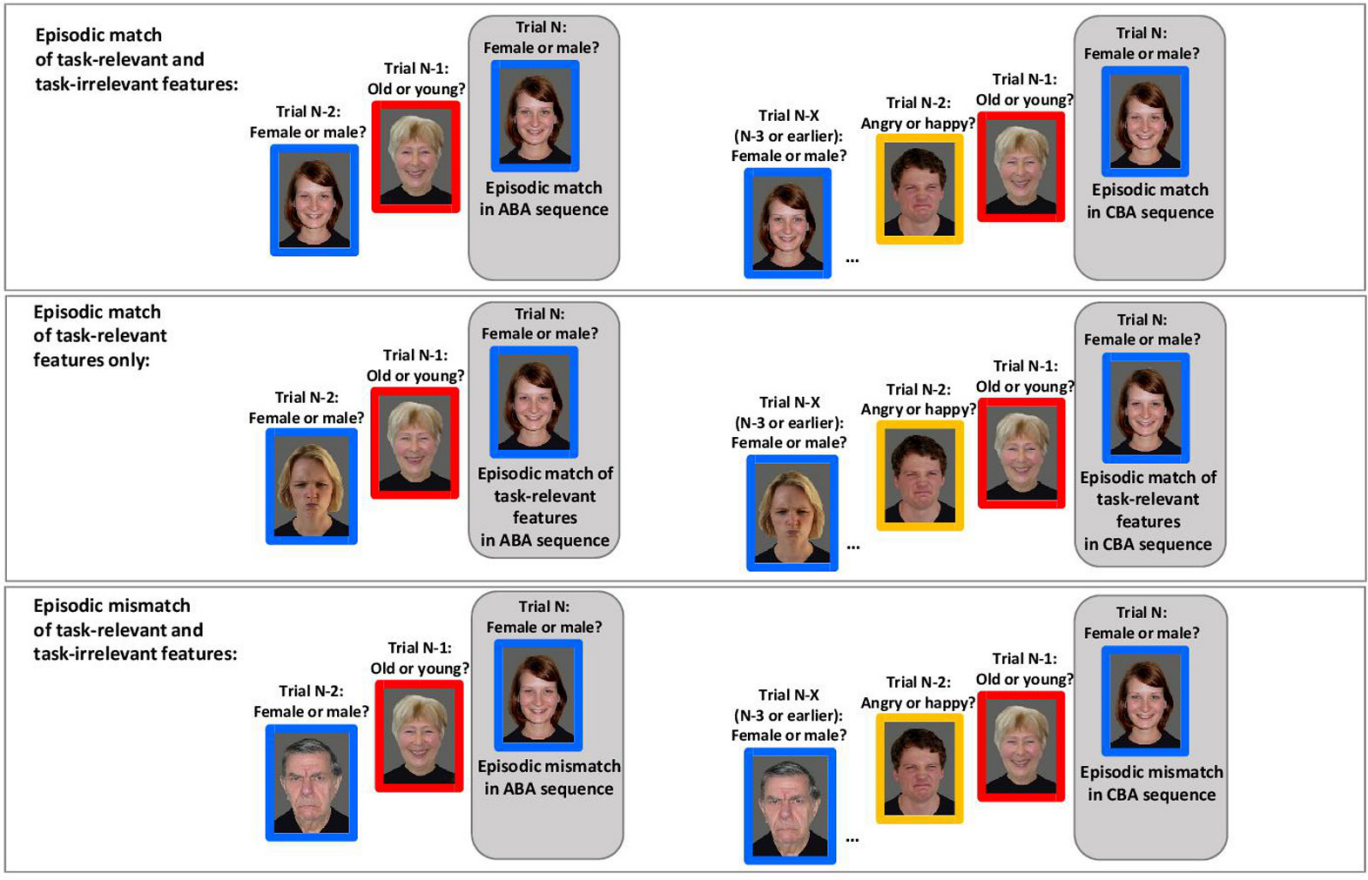
Illustration of the N-X contrast as a method for assessing contributions of episodic interference to N-2 task repetition costs. Episodic interference in the current trial N is defined with respect to the last occurrence of the same task in trial N-2 (in ABA sequences) or in trial N-3, N-4, N-5, etc., summarized as N-X (in CBA sequences). The N-X contrast is shown for three different levels of episodic interference between the current task in Trial N and the most recent episode of that task in Trial N-X: Episodic match of task-relevant and task-irrelevant features (upper row); Episodic match of task-relevant features only (i.e., of stimulus category and associated response; middle row); Episodic mismatch of task-relevant features (stimulus category and associated response) and task-irrelevant features (stimulus identity; lower row). Participants switch between three different face categorization tasks: Indicating whether the presented face is female or male (task cue: blue frame around the picture); indicating whether the face belongs to an old or young person (task cue: red frame); indicating whether the face shows an angry or happy expression (task cue: yellow frame). The stimulus set included 40 different pictures overall (each showing a different person; five different persons for each combination of task-relevant categories, e.g., female-young-happy). Participants responded by pressing a left or right response key; the same two keys were used for all three tasks, and the response mappings were counterbalanced across participants (see text for details).

**The “N-X contrast”.** In order to overcome these potential shortcomings of the “N-2 contrast”, here we suggest an alternative way of assessing episodic interference contributions to N-2 task repetition costs, which we term the “N-X contrast”. As before, N-2 task repetition costs are measured as the performance difference between ABA and CBA task sequences. Other than before, however, episodic interference conditions are now defined with respect to the last occurrence of task A. In ABA task sequences, task A last occurred in trial N-2. In CBA task sequences, task A last occurred in trial N-3 or further back (e.g., ACBA, ABCBA, ACBCBA, etc.). The “N-X contrast” is illustrated in Figure 2.

**Defining episodic matches in categorization tasks.** Mayr’s (2002) paradigm was especially designed for assessing episodic contributions to N-2 task repetition costs, and this paradigm was also used in the work by Grange and colleagues (Grange, 2018; Grange et al., 2017, 2019; Kowalczyk & Grange, 2019). Yet, the question arises whether the results from this paradigm can be generalized to other task-switching paradigms. For one, Mayr’s (2002) paradigm relies heavily on spatial information processing (as all three tasks involve spatial transformation rules), and results from spatial processing might not necessarily generalize to other domains of cognition. Moreover, four response alternatives are used in this paradigm, rendering N-2 response repetitions less likely than N-2 response switches. N-2 response repetitions might thus have been perceived as more salient than N-2 response switches, and the perceived saliency might in turn have speeded up performance in this condition.

Researchers might be interested in re-evaluating the results from previous studies where different task-switching paradigms were used, and the resulting N-2 task repetition costs were interpreted as a marker of task-level inhibition. For instance, Gade et al. (2016), aimed to assess the role of episodic interference effects to N-2 task repetition costs in the data of a previously published study. In that previous study (Gade & Koch, 2014), participants switched between three different categorization tasks: categorizing a letter as consonant or vowel, categorizing a digit as odd or even, and categorizing a symbol as being typically encountered in a text or math context. Gade and Koch (2014) used eight different letters, eight different digits, and eight different symbols, rendering stimulus repetitions from N-2 to N less likely than in the Mayr (2002) paradigm. (In Mayr’s paradigm, only four different stimuli were used, such that N-2 stimulus repetitions occurred in about 25% of the trials.) Moreover, Gade and Koch (2014) presented a compound stimulus in every trial, which consisted of one letter, one digit, and one symbol, arranged in a column. The spatial positions of letter, digit, and symbol in the column varied randomly from trial to trial. Therefore, even when the task-relevant feature of the compound stimulus repeated (e.g., when the relevant task was digit categorization, and the same digit was presented in trials N-2 and N), the task-irrelevant features almost always changed (e.g., the spatial position of the digit could change from N-2 to N, and the identity and spatial position of letter and symbol could change as well). In an attempt to assess episodic retrieval contributions in these data, Gade et al. (2016) compared N-2 task-repetition costs between N-2 stimulus repetitions (where stimulus identity of the task-relevant stimulus feature repeated from N-2 to N) and N-2 stimulus switches (where the identity of the task-relevant stimulus feature did not repeat). In ABA sequences, the N-2 stimulus repetitions defined in this way always involved a N-2 response repetition; the N-2 stimulus switches, on the other hand, could involve either a N-2 response repetition or switch.^4^ Gade et al. (2016) found that N-2 task-repetition costs were reduced or even absent in N-2 stimulus repetitions relative to N-2 stimulus switches, and concluded that episodic interference effects also contributed to the N-2 task-repetition costs reported in Gade & Koch (2014).^5^

In general, if one wishes to assess the contribution of episodic retrieval effects to N-2 task repetitions costs in other task-switching paradigms (not originally designed for this purpose), the situation often becomes more complex. Many task-switching paradigms in the literature involve categorization tasks, where several different stimuli per category are used (e.g., categorization of digits as odd or even; categorization of letters as consonant or vowel, etc., as in Gade & Koch’s paradigm discussed above). Hence, when the task-relevant stimulus feature repeats (e.g., the category), this does not necessarily involve a repetition of the task-irrelevant features (e.g., stimulus identity, spatial position of the stimulus, etc.) The question therefore arises of how to define episodic matches between task episodes – do episodic matches require a match of *all* episodic features, or is a match of the *task-relevant* episodic features enough to produce episodic facilitation effects? The results of the re-analysis reported in Gade et al. (2016) seem to suggest that matching task-relevant episodic features (with mismatching task-irrelevant features) are enough to produce episodic facilitation effects; however, this might depend on the specific paradigm applied, and on the saliency of the task-irrelevant features.

From a theoretical perspective, it seems reasonable to assume that task-relevant features are weighted more strongly than task-irrelevant features in an episodic memory trace (Frings et al., 2020; Memelink & Hommel, 2013). When adopting this assumption, one would predict that a match of the task-relevant features can produce episodic facilitation even if the task-irrelevant features do not match. In task-switching paradigms involving categorization tasks, it thus makes sense to manipulate the degree of episodic interference in three levels: full episodic match (of both task-relevant and task-irrelevant features), episodic match of task-relevant (but not task-irrelevant) features, episodic mismatch of both kinds of features. In the present study, we made the assumption that task-relevant features are being given more weight than task-irrelevant features in an episodic memory representation, and manipulated those three levels of episodic interference in Experiment 1. In the re-analysis of previously published data reported first, we distinguished between two levels of episodic interference (episodic match of task-relevant features versus episodic mismatch), because full episodic matches were excluded by design in those data.

### Aim of present paper

In the present study, we further explore the contribution of episodic interference effects to N-2 task-repetition costs, by using the N-X contrast. In this contrast, episodic interference between task episodes in task-switching is defined with respect to the last occurrence of the same task, which could have occurred several trials back (N-2, N-3, N-4, etc., summarized as N-X). We focus on task-switching paradigms with categorization tasks, as these are widely used in the task-switching literature. In a first step, we re-analyze previously published data, in order to explore to what extent episodic interference contributed to the N-2 task-repetition costs in these data. In a second step, we report a new, pre-registered, experiment with a categorization-tasks paradigm, where we manipulated the degree of episodic interference between task episodes in three levels: a) Full episodic matches, where all features (stimulus category, response, and stimulus identity) repeated between current and previous episode of the same task; b) Episodic matches of the task-relevant features, where the task relevant features (stimulus category and response) repeated, but the task-irrelevant stimulus feature (stimulus identity) did not; c) Episodic mismatches, where both task-relevant features (stimulus category and response) and task-irrelevant features (stimulus identity) differed between current and previous episode of the same task. In addition to analysis of mean performance, we also performed diffusion modeling of the data (see also Kowalczyk & Grange, 2019); the diffusion-model analysis is reported in the Online Supplementary Material.

## Re-analysis of published data

In a first step, we re-analyzed the combined data from two previous studies (Schuch, 2016; Schuch & Konrad, 2017), where we had measured N-2 task-repetition costs and interpreted these as a marker of task-level inhibition. In this re-analysis, we aimed to explore whether episodic interference effects contributed to the N-2 task-repetition costs in those studies. To this end, we compared N-2 task-repetition costs between two different levels of episodic interference. We reasoned that if N-2 task-repetition costs are larger in the condition with more episodic interference than in the condition with less episodic interference, then episodic mechanisms likely contributed to the observed N-2 task-repetition costs.

We first needed to establish which levels of episodic match between task episodes can be distinguished in those data (as these studies were not originally designed for assessing episodic interference effects). In both Schuch & Konrad (2017) and Schuch (2016), participants switched between three face categorization tasks. In each trial, a picture of a face was presented, and participants had to decide whether the face belonged to a young or old person (age categorization task), female or male (gender categorization task), and whether the facial expression was happy or angry (facial expression categorization task). They always responded by pressing one of two keys (left or right). The task switched on every trial, and was always indicated by the color of a frame that was presented at the beginning of the trial. Overall, the pictures of 40 different persons were used, and it was controlled that the person presented in one task episode was not presented again in the next episode of the same task. That is, full episodic matches were excluded by design in these data. However, two levels of episodic interference can be distinguished in these data: The task-relevant stimulus category and response could either match or mismatch between two subsequent episodes of the same task. For instance, when considering two episodes of the gender task, both episodes could involve a female face, which would afford a left key press in both episodes, or one episode could involve a male face affording a right key press and the next episode a female face affording a left key press, such that both stimulus category and response would switch from one episode to the next.

We can thus define two different levels of episodic interference in these data: Episodic matches of task-relevant features (where the stimulus category and the response match) versus Episodic mismatches (where stimulus category and response do not match). One could argue that the category-reponse rule (sometimes also termed “action rule”, “response rule”, or “S-R binding”) constitutes a task-relevant feature in itself, in addition to stimulus category and physical response. For simplicity, we only consider category and response as task-relevant features here; our line of argument would remain the same if the category-reponse rule was considered as an additional feature.

In order to address our research question whether the observed N-2 task-repetition costs are due to task-level inhibition and/or episodic interference, we compared the size of N-2 task-repetition costs between the two different levels of episodic interference (i.e., Episodic mismatches versus Episodic matches of task-relevant features). We reasoned that if N-2 task-repetition costs were larger in Episodic mismatches than in Episodic matches of task-relevant features, this would indicate a contribution of episodic mechanisms to the observed N-2 task-repetition costs. If, on the other hand, N-2 task-repetition costs did not differ between the two episodic interference conditions, this would indicate that these costs are driven by a task-level inhibitory mechanism.

### Method

**Young adults’ groups from Schuch & Konrad (2017) and Schuch (2016).** The young adults’ groups from the studies by Schuch & Konrad (2017) and Schuch (2016) were combined, resulting in a sample of 56 participants in total (mean age = 22.3 years, sd = 2.6, range 18–30 years; 24 women and 32 men).^6^

**Paradigm used in Schuch & Konrad (2017) and Schuch (2016).** Portrait photographs of 40 adults were used as stimulus material. 20 of those adults were female and the other 20 were male. Furthermore, half of them were younger adults (20 to 30 years) and the other half elderly adults (60 to 70 years), and each photographed person either showed a happy or an angry facial expression (see Schuch et al., 2012, for further details regarding the stimulus material). The portrait photographs always appeared inside a colored frame, which varied between red, blue, and yellow. When frame color was red, the participants had to indicate whether the person on the photograph inside the frame was young or old (age task). A blue frame required participants to decide whether the person they saw was male or female (gender task). The yellow frame indicated to categorize the person as looking happy or angry (facial expression task). Participants responded by pressing a left or right response key (the “x” and “,” keys on a German QWERTZ computer keyboard) with their index fingers. Respone mappings were counterbalanced across participants, with half of the participants responding with the left key to happy, young, and male faces, and with the right key to angry, old, and female faces; the other half of participants received the reverse mapping. Every trial started with the presentation of a red, blue, or yellow frame for 500 ms, followed by the presentation of a photograph inside the frame. The frame and picture stayed on the screen until the left or right response key was pressed. Then, the screen turned black for 1,000 ms. In case of a wrong response, an error feedback appeared. Trials were presented in pseudorandom order, with the constraints that every trial was a task switch, and the person presented in a particular trial n was never the same as the persons presented in trials n-1 and n-2 (see Schuch & Konrad, 2017, for a full list of constraints of the pseudorandom sequence). Participants performed a practice session, followed by 4 blocks of 60 trials each, separated by short breaks.

**Design.** For the N-X contrast, a 2 × 2 within-subject design was applied with the independent variables N-2 Task Transition (ABA, CBA) and Episodic Match Condition (Match of task-relevant features: stimulus category and response match between current episode and most recent episode of the same task; Mismatch of task-relevant features: Different stimulus categories and responses in current and most recent episode of the same task; the task-irrelevant feature stimulus identity always differed between current and previous episode of the same task). The dependent variables were RT and error rates.

In the Online Supplementary Material, we also report the N-2 contrast, where a 2 × 2 within-subject design was applied with the independent variables N-2 Task Transition (ABA, CBA) and N-2 Response Transition (N-2 response repetition vs N-2 response switch). The N-2 contrast differs from the N-X contrast with respect to the CBA conditions: in the N-2 contrast, the episodic interference conditions are defined with respect to the N-2 trial, and two conditions can be distinguished: Partial match of features (i.e., the response, but not the task and stimulus category, match between trial N and trial N-2) versus Mismatch of all features (response, task, and stimulus category all change between trial N and trial N-2).

### Results and Discussion

**Distribution of task lags.** We first checked the distribution of task lags in CBA trials. In total, there were 111.3 CBA trials per participant on average (minimum [min] 104, maximum [max] 117). Of these, 48.6 trials on average (min 45, max 53) were N-3 task repetitions; 30.1 trials (min 29, max 31) were N-4 task repetitions; 17.1 trials (min 15, max 19) were N-5 task repetitions; 15.4 (min 14, max 17) were N-6, N-7, or N-8 task repetitions. In ABA trials (where all trials were N-2 task repetitions), there was an average of 114.1 trials per participant (min 113, max 119).

**Data filtering.** The first two trials from each experimental block, the two trials following an error, and outliers (trials with RT larger than three standard deviations (sd) from a participants’ overall mean RT) were excluded. For RT analysis, error trials were excluded as well. While N-2 stimulus repetitions were excluded by design (see above), stimulus repetitions with a larger lag occurred in a small number of trials (i.e., trials where the person presented in the current task episode was the same as the person presented in the last episode of that task in trial N-3 or further back); these trials were excluded from analysis, leading to exclusion of 1.7% of trials per participant on average (sd 0.3%, min 0.6%, max 2.2%). The mean number of trials per condition included in the analysis is summarized in Table S1 in the Online Supplemental Material.

**Figure 3:**
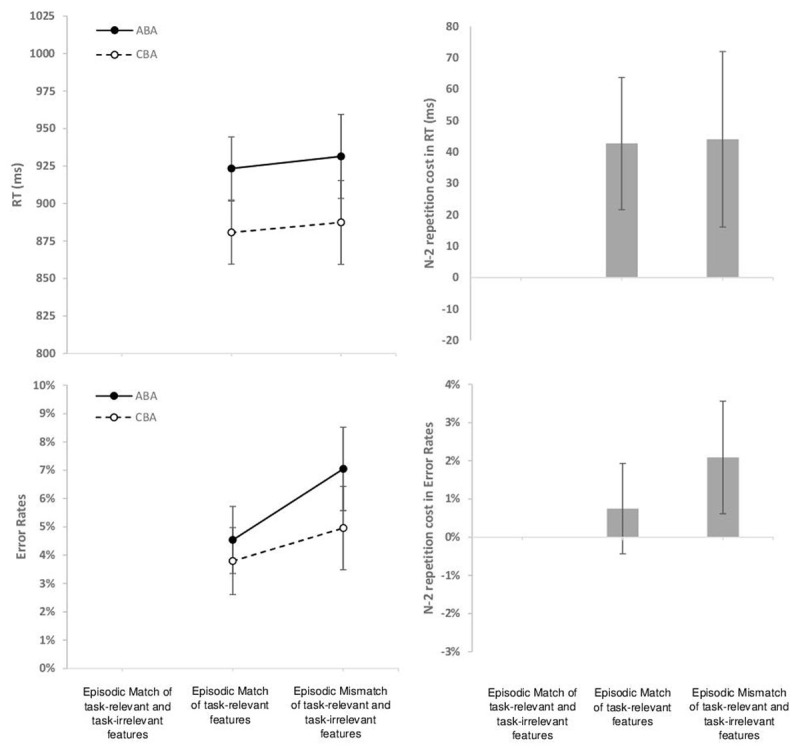
Re-analysis of the young adults’ groups from Schuch & Konrad (2017) and Schuch (2016). N = 56. The condition of Episodic Match of task-relevant and task-irrelevant features was not manipulated in these data. Left side: Mean RT (upper row) and mean Error Rate (lower row) as a function of N-2 Task Transition (N-2 Task Repetition [ABA] versus N-2 Task Switch [CBA]) and Episodic Match Condition (Episodic Match of task-relevant features [stimulus category and response] between current and last task episode; Episodic Mismatch: Switch of the task-relevant features [stimulus category and response] between current and last task episode). Right side: N-2 task-repetition costs as a function of Episodic Match Condition in RTs and Error Rates. Error bars represent the 95% confidence interval of the ABA-CBA difference per Episodic Match Condition (Pfister & Janczyk, 2013).

**N-X contrast.** The descriptive data are presented in Figure 3. In RT, the 2 × 2 within subject ANOVA with the independent variables N-2 Task Transition and Episodic Match Condition revealed a main effect of Task Transition, *F*(1,55) = 33.50, *p* < .01, *η*^*2*^_*p*_ = .38, indicating overall N-2 task-repetition costs. There was no main effect of Episodic Match Condition, and no interaction, *F*s(1,55) < 1. For exploratory purposes, we computed t-tests assessing N-2 repetition costs separately for the two episodic match conditions; N-2 task repetition costs were 42 ms (standard error of mean [sem] 11 ms, *t*(55) = 3.80, *p* < .01, *d* = 0.51) in episodic matches of the task-relevant features, and 43 ms (sem 14 ms, *t*(55) = 3.05, *p* < .01, *d* = 0.41) in episodic mismatches.

In Error Rates, the respective 2 × 2 ANOVA also yielded a main effect of Task Transition, *F*(1,55) = 11.09, *p* < .01, *η*^*2*^_*p*_ = .17, again indicating overall N-2 task-repetition costs. There was also a main effect of Episodic Match Condition, *F*(1,55) = 16.54, *p* < .01, *η*^*2*^_*p*_ = .23, the latter indicating higher error rates in episodic mismatches than episodic matches of task-relevant features. Again, there was no significant interaction, *F*(1,55) = 1.38, *p* = .25, *η*^*2*^_*p*_ = .03. When analyzed separately in exploratory t-tests, N-2 task repetition costs were 0.9% (sem 0.6%, *t*(55) = 1.43, n.s., *d* = 0.20) in episodic matches, and 2.0% (sem 0.7%, *t*(55) = 2.74, *p* < .01, *d* = 0.39) in episodic mismatches.

**Discussion of N-X contrast.** We re-analyzed the dataset from Schuch & Konrad (2017) and Schuch (2016), investigating whether N-2 task-repetition costs differ between different episodic interference conditions. We compared a condition where the task-relevant features (i.e., stimulus category and response) were the same as in the last episode of the same task with another condition where the task-relevant features (stimulus category and response) differed from the last episode of the same task (- note that in these data, the task-irrelevant feature [stimulus identity] always changed from one episode to the next episode of the same task).

In RT data, we did not observe any evidence that performance differed between the two episodic interference conditions: N-2 task-repetition costs were of very similar size in both conditions (around 40 ms), as was the absolute mean RT in the ABA and CBA conditions. In error data, N-2 task-repetition costs were numerically larger in the episodic mismatch condition than episodic match condition (the costs were about 2% versus 1%), but the interaction was not statistically significant. Thus, the descriptive data pattern is consistent with the possibility that episodic interference might have contributed to the N-2 task-repetition costs in error rates, although this possibility was not statistically corroborated (- possibly, the interaction would be significant with a larger sample size than N = 56; see Grange et al., 2017).

Interestingly, there was also a main effect of Episodic Condition: mean error rates were larger in episodic mismatches than episodic matches (when averaged across ABA and CBA condition). This main effect suggests that when switching back to a task (after one or more intermediate trials), repeating the task-relevant features of the previous task episode facilitates performance relative to switching all task features.

## Experiment 1

This new experiment was designed to assess in more detail the potential contributions of episodic interference to N-2 repetition costs in the categorization-tasks paradigm used by Schuch & Konrad (2017) and Schuch (2016). We additionally introduced a condition of full episodic match between previous and current task episode (i.e., a repetition of response, stimulus category, *and* stimulus identity). Also, we considerably increased the number of trials per condition, which is one way of increasing statistical power (e.g., Brysbaert, 2019; Kolossa & Kopp, 2018; Rouder & Haaf, 2018).

We pre-registered the following predictions: If task inhibition was the only underlying mechanism for N-2 task repetition costs, one would expect N-2 task repetition costs of the same size across all three episodic match conditions (because the level of episodic interference should not affect N-2 task repetition costs). If, on the other hand, episodic interference was the only underlying mechanism for N-2 task repetition costs, one would expect N-2 task repetition costs for episodic mismatches (because there would be episodic interference in both ABA and CBA trials, and assuming that episodic memory traces decay over time, one would expect more episodic interference in ABA than CBA trials), and possibly one would expect N-2 task repetition costs also for the condition where task-relevant features match (for the same reasons). Crucially, if episodic interference was the only underlying mechanism for N-2 task repetition costs, one would not expect any N-2 task repetition costs for full episodic matches; if anything, one might expect a N-2 task-repetition *benefit* in this condition (because there would be episodic facilitation in both ABA and CBA, and assuming that episodic memory traces decay over time, such episodic facilitation might be stronger in ABA than CBA).

Finally, if both task inhibition and episodic interference contributed to N-2 task repetition costs, one would expect N-2 task-repetition costs across all three episodic conditions, but these N-2 task-repetition costs should differ in size: They should be smaller for full episodic matches than for the other two conditions (because in full episodic matches, the two mechanisms would work in opposite directions, whereas in the other conditions, they would work in the same direction). We also hypothesized that N-2 task-repetition costs would differ between the other two conditions, and would be smaller for the condition with episodic matches of task-relevant features than for the condition with episodic mismatches (- this latter hypothesis was based on the observed data pattern in error rates in the re-analysis reported above, which is consistent with the assumption that task-relevant features are weighted more strongly than task-irrelevant features).

### Method

**Pre-registration.** This experiment was pre-registered at https://aspredicted.org/iw63x.pdf.

**Participants.** 40 participants (29 female and 11 male) took part in the experiment (mean age = 23.08 years, sd = 2.94; range = 18 – 30). They were students, or friends of students, at RWTH Aachen University. 30 participants received monetary compensation (8 Euros per full hour) or partial course credits; 10 participants did not receive any compensation.

**Sample size and statistical power.** The pre-registered sample size of N = 40 was determined by our aim to keep Experiment 1 as similar as possible to the earlier studies. We therefore used the same stimulus set as before, which consisted of N = 40 stimuli overall. We divided this stimulus set into five different subsets of eight stimuli each (- note that eight stimuli is the minimum number of stimuli needed per participant when using a paradigm where participants switch between three different categorization tasks). Each participant received one of the five different subsets. We additionally fully counterbalanced the eight possible response mappings across participants, resulting in 5 × 8 = 40 different combinations of stimulus set and response mapping.

We were mainly interested in the 2 × 3 within-subject interaction of our design with the independent variables N-2 Task Transition and Episodic Match Condition (see Design section below). We did not know a priori what effect size to expect for this interaction. Using the MorePower software version 6.0.4 (Campbell & Thompson, 2012), we calculated that with a sample size of N = 40, and power of .80, we would be able to detect an interaction with effect size of *η*^*2*^_*p*_ = .11, corrresponding to a medium-to-large effect. It is thus possible that we would miss an interaction with a smaller effect size with our sample size. On the other hand, we used a relatively large number of trials in Experiment 1 (92 to 404 trials per participant and condition for RT analysis; see Table S1 in Online Supplemental Material). Increasing the number of trials per condition is one way of increasing statistical power, particularly in within-subject designs (e.g., Brysbaert, 2019; Kolossa & Kopp, 2018). The reason is that the larger the number of observations per cell of the design, the higher the precision with which the means per conditions can be estimated, i.e., the higher the reliability of the dependent measure. Rouder and Haaf (2018) demonstrated that larger trial numbers increase statistical power considerably when the population-level variability of the effect of interest is small relative to the trial-by-trial variability in the experiment, as is probably the case for N-2 task repetition costs in RTs in a task-switching paradigm.

**Tasks, stimuli, and responses.** The paradigm was the same as in the studies by Schuch & Konrad (2017) and Schuch (2016). In order to make stimulus repetitions more likely, the overall set of 40 stimuli was divided into five subsets of eight stimuli each, such that each subset contained one stimulus for each combination of the attributes age (old vs young), gender (female vs male), and emotional expression (happy vs angry). The stimulus set was counterbalanced across participants; one participant received only one subset of stimuli. As in the previous studies, participants used a left and right key for responding (the “x” and “,” keys on a QWERTZ keyboard, which they pressed with their left and right index finger). The eight possible combinations of response mappings were fully counterbalanced across participants. Response mappings and stimulus subset were counterbalanced orthogonally, resulting in 8 × 5 = 40 different combinations (one unique combination of stimulus subset and response mapping for each participant).

**Procedure.** The overall duration of the experiment was around two hours. Participants performed the experiment either in a laboratory room at RWTH Aachen University, or (due to onset of the Covid-19 pandemic) at home using their own computer or laptop. The experiment was programmed using the software Presentation (https://www.neurobs.com), and the Presentation Package Player software was used for the home testing sessions (this software only runs under a Windows operating system). Participants received instructions either in-person (when tested in the lab) or via video-conferencing (when tested at home). Instructions were administered both orally and in written format on the computer screen. Participants were encouraged to answer as quickly as possible and as accurately as possible. The experiment started with four practice blocks with 16 trials each. In the first three of these practice blocks, participants practiced one of the three tasks individually, starting with the age task, followed by the gender task and lastly the facial expression task. In the fourth practice block, all three tasks were intermixed.

The experiment consisted of 16 experimental blocks of 120 trials each, separated by self-paced breaks after each block. Participants were informed when half of the experiment was completed. Task cues and stimuli occurred pseudo-randomly with the following constraints: Every trial was a task switch, and N-2 task repetitions and N-2 task switches occurred approximately equally often in each block. Moreover, the three tasks also occurred equally often (40 times each) per block, and each combination of a particular stimulus and task appeared 5 times per block. The number of N-2 response repetitions and N-2 response switches was approximately the same within each block of trials, as was the number of N-1 response repetitions and N-1 response switches.

The trial procedure was the same as in Schuch & Konrad (2017) and Schuch (2016): At the beginning of each trial, a red, blue, or yellow frame was presented for 500 ms, indicating the task that was to be performed. Subsequently, a portrait photograph appeared inside the colored frame, and the screen remained unchanged until a response was given. Afterwards the screen turned black for 1,000 ms until the next trial started. In case of an incorrect response, an error feedback occurred after 500 ms, lasting 1,000 ms. Thereafter, the error feedback disappeared, and the screen stayed black again for another 500 ms before the next trial started.

**Design.** A 2 × 3 within-subject design was applied with the independent variables N-2 Task Transition (ABA, CBA) and Episodic Match Condition (Full episodic match; Episodic match of task-relevant features; Episodic mismatch). The dependent variables were RT and error rates; separate 2 × 3 ANOVAs were conducted for each dependent variable. In case of violation of the sphericity assumption (as indicated by a significant Mauchly’s test for sphericity, *p* < .05), the Greenhouse-Geisser adjustment was applied and the corresponding *ε* value for correction of the degrees of freedom (dfs) is reported.

In the Online Supplementary Material, we also report the corresponding N-2 contrast, where a 2 × 3 within-subject design was applied with the independent variables N-2 Task Transition (ABA, CBA) and N-2 Stimulus/Response Transition (N-2 repetition of stimulus and response, N-2 repetition of response only, N-2 switch of stimulus and response).

### Results and Discussion

**Distribution of task lags.** The distribution of lags in CBA trials was as follows: In total, there were 930.3 CBA trials per participant on average (min 668, max 941). Of these, 438.2 trials (min 316, max 443) were N-3 task repetitions; 253.8 trials (min 182, max 257) were N-4 task repetitions; 122.1 trials (min 88, max 123) were N-5 task repetitions; 116.3 (min 82, max 118) were N-6 to N-10 task repetitions. In ABA trials (where all trials were N-2 task repetitions), there was an average of 932.9 trials per participant (min 666, max 943).

**Data filtering.** Data filtering was the same as for the re-analysis reported above, and as specified in the pre-registration. In a small number of trials, participants received an error feedback despite a correct response due to programming error; these and the immediately following trials were excluded from data analysis (affecting about 0.2% of the trials). For one participant, only the data from 11 of the 16 experimental blocks were available, because the computer crashed during block 12 (see Table S1 for a summary of the average number of trials per experimental condition).

**Figure 4:**
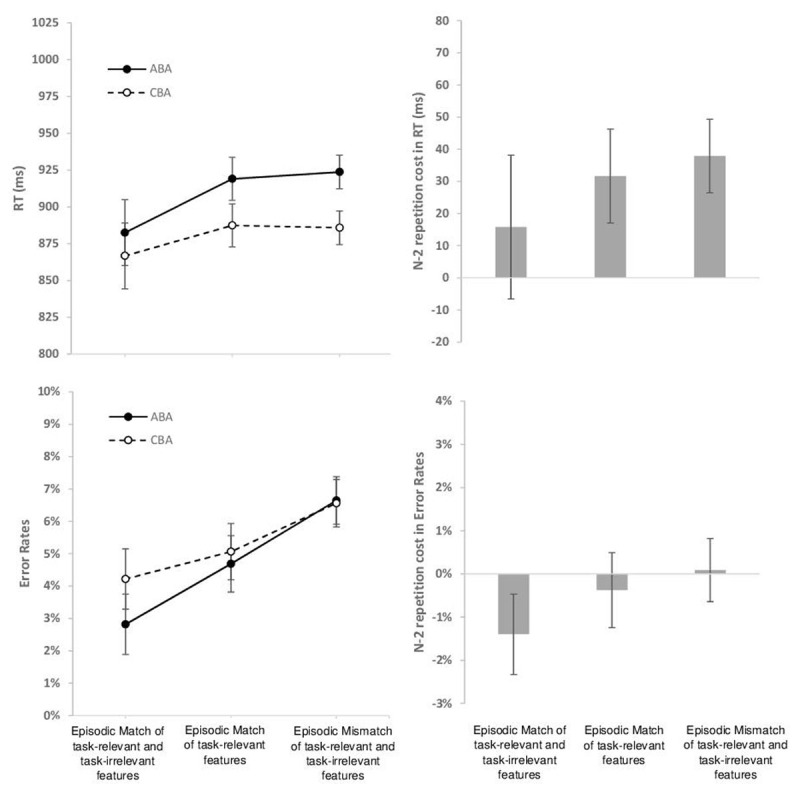
Experiment 1. N = 40. Left side: Mean RT (upper row) and mean Error Rate (lower row) as a function of N-2 Task Transition (N-2 Task Repetition [ABA] versus N-2 Task Switch [CBA]) and Episodic Match Condition (Full Episodic Match: Repetition of all task features [stimulus identity, stimulus category, and response] between current and last task episode; Episodic Match of task-relevant features [stimulus category and response]; Episodic Mismatch: Switch of the task-relevant features [stimulus category and response]). Right side: N-2 task-repetition costs as a function of Episodic Match Condition in RTs and Error Rates. Error bars represent the 95% confidence interval of the ABA-CBA difference per Episodic Match Condition (Pfister & Janczyk, 2013).

**N-X contrast.** The descriptive data are shown in Figure 4. The ANOVA on RTs disclosed a significant main effect of N-2 Task Transition *F*(1, 39) = 26.73, *p* < .001, *η*^*2*^_*p*_ = .41, indicating N-2 task repetition costs, and a significant main effect of Episodic Match Condition, *F*(2, 78) = 18.60, *p* < .001, *η*^*2*^_*p*_ = .32, indicating slower RTs with increasing mismatching features. The two-way interaction was not significant, *F*(2, 78) = 2.15, *p* = .14, *ε* = 0.71, indicating that N2 task repetition costs did not statistically differ between the three episodic conditions. When analyzed separately in exploratory post-hoc t-tests, N-2 task repetition costs were significant in the mismatch condition (mean = 38 ms, SEM = 6 ms, *t*(39) = 6.62, *p* < .001, *d* = 1.05) and the condition of episodic match of task-relevant features (mean = 32 ms, SEM = 7 ms, *t*(39) = 4.33, *p* < .001, *d* = 0.68), but not in the full episodic match condition (mean = 16 ms, SEM = 11 ms, *t*(39) = 1.41, *p* = .17, *d* = 0.22).

The corresponding ANOVA on error rates revealed a different pattern than the RT analysis: There was a significant main effect of N-2 Task Transition, *F*(1, 39) = 7.14, *p* = .011, *η*^*2*^_*p*_ = .16, indicating a N-2 task repetition *benefit* across all conditions. There was also a significant main effect of Episodic Match Condition, *F*(2, 78) = 35.25, *p* < .001, *η*^*2*^_*p*_ = .48, *ε* = 0.75, indicating that error rates became higher with increasing mismatching features. The two-way interaction was not significant, *F*(2, 78) = 2.82, *p* = .065. When analyzed separately for the different episodic conditions in exploratory post-hoc t-tests, N-2 task repetition costs were not significant in the mismatch condition (mean N-2 task repetition cost = 0.09%, SEM = 0.37%, *t*(39) < 1, *d* = 0.04), and neither in the condition of episodic match of task-relevant features (mean = –0.38%, SEM = 0.44%, *t*(39) < 1, *d* = 0.14); in the condition of full episodic matches, a significant negative N-2 repetition cost (i.e., N-2 repetition benefit) was obtained (mean = –1.40%, SEM = 0.47%, *t*(39) = 3.00, *p* = .005, *d* = 0.47).

**N-X contrast when excluding all task sequences with previous errors.** In the Online Supplemental Material, we report two additional analyses of the N-X contrast where we used different filtering criteria. In the first additional analysis, we additionally excluded all CBA sequences in which participants had made an error during the last episode of task A (which had occurred in trial N-3 or further back). The results were virtually identical to those reported above, suggesting that including or excluding these trials with an erroneous previous task episode did not change the result pattern.

In the second additional analysis, we controlled for intermediate errors that might have occurred between the last and the current episode of task A in CBA sequences (e.g., in a ABCBA task sequence, an error might have occurred while performing task B in trial N-3). To this end, we restricted the CBA sequences to only include N-3, N-4, and N-5 task repetitions (i.e., only task sequences of the types ACBA, ABCBA, and ACBCBA, which included about 87% of all CBA sequences). Moreover, we excluded *five* trials after each error (instead of only *two* trials after each error as in the main analysis reported above); this means that all included trials were preceded by at least five previous correct trials. The result pattern was again similar as before, with the exception that we now observed significant N-2 task repetition costs in the full episodic match condition in RTs. Hence, taken together, the results were similar when controlling for previous errors.

**Discussion of N-X contrast.** In the new experiment, we unexpectedly observed a diverging data pattern in RTs and error rates: While N-2 task repetition *costs* were obtained in RTs, N-2 task repetition *benefits* were observed in error rates. For this reason, the results are difficult to interpret with respect to the research question of whether N-2 task repetition costs reflect inhibition or episodic interference.

We did not observe a significant modulation of N-2 repetition costs by episodic condition. In order to further explore the data pattern, we performed exploratory post-hoc t-tests on N-2 task repetition costs in the different episodic interference conditions. In the condition of full episodic matches, N-2 task repetition costs in RTs were not significant, except in an additional analysis when controlling for previous errors in the task sequence. In error rates, a significant N-2 task repetition benefit was observed with full episodic matches. When some features (task-irrelevant or task-relevant) mismatched between current and previous episode of the same task, there were significant N-2 task repetition costs in RTs (and no costs or benfits in error rates). The data pattern seems consistent with the possibility that both inhibition and episodic intereference contribute to N-2 task repetition costs, but is not fully conclusive in this respect.

A consistent result was that overall performance (averaged across ABA and CBA) became worse with increasing episodic mismatch between current and previous episode of the same task (- the corresponding main effect was significant in both RTs and error rates). Hence, the degree of episodic interference between current and previous episode of the same task influenced performance, regardless of the lag between those task episodes (lag = 2 in ABA condition, lag > 2 in CBA condition). In order to further explore the influence of lag between task episodes, we performed an additional analysis where we split the CBA data into lag3 (i.e., ACBA), lag4 (i.e., ABCBA), lag5 (i.e., ACBCBA), and lag6 or larger (i.e., A…BCBCBA).

The inhibition and episodic interference accounts make different predictions for this additional task-lag analysis: The inhibition account assumes that persisting task-level inhibition decays over time (or as a function of intermediate tasks or trials), and hence predicts that performance improves with increasing task lag in all episodic conditions. The episodic interference account, if it assumes that episodic memory traces decay over time, would predict a differential pattern: for episodic mismatches, one would expect performance to improve with increasing lag, but for full episodic matches, one would expect performance to decline with increasing lag. If decay of episodes over time is not assumed, one would not predict any effect of lag on performance.

**Figure 5:**
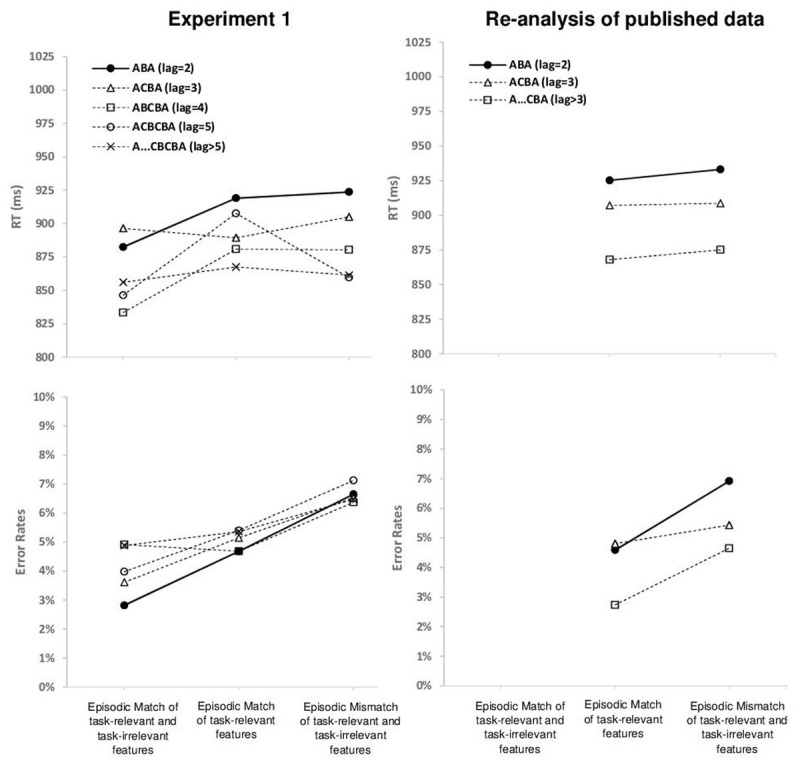
Mean RT (upper row) and mean Error Rate (lower row) as a function of Task Lag and Episodic Match Condition. In Experiment 1, five different levels of Task Lag were distinguished (left side); in the re-analysis of the published data, three different levels of Task Lag were distinguished due to lower trial numbers (right side). Error bars are not shown for better visualization of the data pattern; see Table S3 in Online Supplemental Material for the standard error of mean per condition.

**Exploratory analysis of performance as a function of task lag.** For Experiment 1, a large number of trials was available per participant, which allowed us to distinguish five different levels of Task Lag: ABA (lag = 2), ACBA (lag = 3), ABCBA (lag = 4), ACBCBA (lag = 5), and A…CBCBA (lag > 5). A 3 × 5 ANOVA with the independent variables Episodic Match Condition and Task Lag was computed; the mean number of trials per participant and condition for RT analysis was 64.6 (min 7, max 217). The descriptive data are presented in Figure 5 (and Table S3 in the Online Supplemental Material). The ANOVA on RT revealed a main effect of Task Lag, *F*(4, 156) = 6.31 *p* < .01, *η*^*2*^_*p*_ = .14, *ε* = .62, indicating that RTs decreased monotonically with increasing task lag: Mean RT for task repetitions with lag2, lag3, lag4, lag5, and lag > 5 was 914 ms, 898 ms, 876 ms, 873 ms, and 862 ms, respectively. There was also a main effect of Episodic Match Condition, *F*(2, 78) = 6.78, *p* < .01, *η*^*2*^_*p*_ = .15, indicating that RTs increased with increasing episodic mismatch. The interaction was not significant, *F*(8, 312) = 1.59 *p* = .19, *η*^*2*^_*p*_ = .04, *ε* = .40. In the ANOVA on error rates, there was no main effect of Task Lag, *F*(4, 156) = 1.42 *p* = .24, *η*^*2*^_*p*_ = .04, *ε* = 0.69, and no interaction, *F*(8, 312) = 1.11 *p* = .35, *η*^*2*^_*p*_ = .03, *ε* = 0.68, but only a main effect of Episodic Match Condition, *F*(2, 78) = 21.12, *p* < .01, *η*^*2*^_*p*_ = 0.35, *ε* = 0.86, indicating that error rates increased with increasing episodic mismatch.

For the previously published data, fewer trials per participant were available; we therefore split the CBA trials into lag3 and lag > 3 CBA trials. The mean number of trials per participant and CBA condition for RT analysis was 23.3 (min 14, max 33). The ANOVA on RT with the independent variables Episodic Match Condition and Task Lag (lag2, lag3, lag > 3) revealed a main effect of Task Lag, *F*(2, 110) = 19.02, *p* < .01, *η*^*2*^_*p*_ = .26, indicating that RTs decreased monotonically with increasing task lag: Mean RT for task repetitions with lag2, lag3, and lag > 3 was 929 ms, 908 ms, and 872 ms, respectively. The main effect of Episodic Match Condition was not significant, *F*(1, 55) < 1, and neither was the interaction, *F*(2, 110) < 1.

The ANOVA on error rates also revealed a main effect of Task Lag, *F*(2, 110) = 8.79, *p* = .01, *η*^*2*^_*p*_ = .14, indicating that error rates decreased monotonically with increasing task lag: Mean error rates for task repetitions with lag2, lag3, and lag > 3 were 5.7%, 4.9%, and 3.6%. There was a main effect of Episodic Condition, *F*(1, 55) = 11.66, *p* < .01, *η*^*2*^_*p*_ = 0.18, indicating that error rates increased with increasing episodic mismatch, and no interaction, *F*(2, 110) = 1.16, *p* = .31, *η*^*2*^_*p*_ = .02, *ε* = 0.79.

**Discussion of task lag analysis.** We observed that RTs decreased monotonically with increasing task lag both in Experiment 1 and in the previously published data. In the latter, the corresponding effect was observed in error rates as well. This main effect of task lag did not interact with Episodic Condition; the descriptive data presented in Figure 5 seem to suggest that the performance improvement with increasing task lag occurred across all episodic conditions (- although the data are somewhat noisy, and the lack of an interaction is always difficult to interpret). The overall performance improvement with increasing task lag is consistent with the idea that persisting task-level inhibition decays as a function of time (and/or as a function of intermediate tasks or trials). This effect cannot be explained by episodic interference: Episodic interference accounts, if they assume that episodic memory traces decay over time, would predict a differential pattern: for episodic mismatches, one would expect that performance improves with increasing lag, but importantly, for full episodic matches, one would expect that performance *declines* with increasing lag. If decay of episodes over time is not assumed, one would not predict any effect of lag on performance. The current finding that performance improves as a function of increasing task lag is consistent with the notion of task-level inhibition that decays slowly over the course of several trials, and can thus be taken as an indicator of task-level inhibition.

## General Discussion

### Summary of present study

The present paper examined to what extent a prominent effect in task switching, namely N-2 task repetition costs, reflects inhibition of task sets or episodic interference between task episodes. Building up on recent theoretical frameworks of episodic binding and retrieval effects (Frings et al., 2020; Hommel, 2004, 2019, Schmidt et al., 2020), we assumed that the task cue – which is usually the first stimulus occurring in a task episode – triggers re-activation of the previous episode of the same task. In task switching, when the task changes on every trial, this previous episode may have occurred in trial N-2, trial N-3, trial N-4, etc. (summarized here as trial N-X). Hence, we defined the degree of episodic interference in task switching with respect to the last episode of the same task (in trial N-X). Further, we distinguished between three levels of episodic interference between two episodes of the same task: Episodic match of both task-relevant and task-irrelevant features; Episodic match of task-relevant, but not task-irrelevant features; Episodic mismatch of both task-relevant and task-irrelevant features. We reasoned that task-relevant features might be weighted more strongly than task-irrelevant features in the episodic memory trace, which could lead to stronger episodic interference effects when task-relevant features mismatch than when task-irrelevant features mismatch (Frings et al., 2020; Memelink & Hommel, 2013).

N-2 task-repetition costs in task switching are computed as the performance difference between lag2 task repetitions (i.e., ABA task sequences) and lag > 2 task repetitions (i.e., CBA task sequences). When participants switch between three different tasks, and immediate task repetitions are not allowed, CBA sequences can be sequences of the following types: ACBA, ABCBA, ACBCBA, etc. While usually, CBA task sequences are not further divided, here we split CBA task sequences into lag3, lag4, lag5, and lag > 5 task repetitions, in order to analyze whether performance depends on the task lag (- note that this part of the analysis was not pre-registered). If a task becomes inhibited when switching away from it, and this inhibition decays slowly over the course of several trials (as is commonly assumed when taking N-2 task repetition costs as a measure of task-level inhibition), then one would expect better performance with increasing task lag across all episodic conditions. Episodic interference accounts, on the other hand, would predict a different data pattern: When assuming that episodic memory traces decay over time, such accounts would predict *better* performance with increasing task lag only for the episodic mismatch condition, but *worse* performance with increasing task lag for the episodic match conditions. If decay of episodes over time is not assumed, episodic accounts would not predict any effect of task lag on performance.

We first re-analyzed previously published data, where two levels of episodic interference could be distinguished: Episodic match versus mismatch of the task-relevant features (- the task-irrelevant features always changed in this data set). We reasoned that if N-2 task repetition costs largely reflected interference between subsequent task episodes, the costs should be larger for episodic mismatches than matches. In the re-analyzed data set, participants switched between three different categorization tasks, and responded to all three tasks with the same two response keys. The stimulus category and the associated response either repeated or did not repeat between two subsequent episodes of the same task (- while stimulus identity never repeated). We found N-2 task repetition costs of similar size when the task-relevant features (i.e., stimulus category and response) matched or did not match between previous and current task episode, suggesting that episodic interference of task-relevant features did not heavily influence the size of N-2 task-repetition costs in this data set. Yet, while we did not observe any significant modulation of N-2 task repetition costs with a sample size of N = 56, the descriptive data pattern in error rates showed numerically larger costs in mismatches than matches, raising the possibility that some modulation might be observed with a larger sample size, which would point to a contribution of episodic interference to N-2 task repetition costs.

In a new, pre-registered, experiment, we included a third episodic interference condition, where both task-irrelevant (stimulus identity) and task-relevant (stimulus category and response) features matched between two subsequent episodes of the same task (full episodic match condition). We reasoned that if N-2 task repetition costs (at least partly) reflected interference between subsequent task episodes, the costs should be largest in the case of episodic mismatch between task episodes, intermediate in the case of episodic match of task-relevant features, and smallest in the case of a full episodic match between task episodes. In RT data, we did not observe any significant modulation of N-2 task repetition costs as a function of episodic interference condition with a sample size of N = 40; numerically the N-2 task repetition costs were largest in the case of episodic mismatch between task episodes, and smallest in the case of a full episodic match. In error rates, we unexpectedly observed N-2 task repetition *benefits* rather than costs, which again were not significantly modulated by episodic interference condition. Numerically, these benefits were most pronounced in the full episodic match condition, and close to zero in the other two conditions. In sum, the results from the new experiment are not fully conclusive, due to the diverging data pattern of N-2 task repetition *costs* in RT and N-2 task repetition *benefits* in error rates. These costs and benefits were not significantly modulated by episodic interference condition; on a descriptive level, the costs in RT were smallest, and the benefits in error rate largest, in the condition of full episodic matches. The descriptive data pattern might be taken as preliminary evidence for a contribution of episodic interference effects to N-2 task repetition costs. In sum, the pre-registered analysis is not fully conclusive with respect to the research question of whether N-2 task repetition costs reflect inhibition or episodic interference.

An important and new result was that overall performance (averaged across ABA and CBA) became worse with increasing episodic mismatch between current and previous episode of the same task (i.e., from “full episodic match” to “match of task-relevant features” to “full mismatch”); this effect consistently occurred in both RTs and error rates (- it was also reflected in diffusion model parameters: drift rate and boundary separation increased, while non-decision time decreased, with increasing episodic mismatch; see Online Supplemental Material). That performance monotonically decreased from the conditions of “full episodic match” to “match of task-relevant features” as an intermediate level, to “episodic mismatch”, corroborates our assumption that the degree of episodic interference between task episodes can be operationalized in this way. These three levels of episodic interference can be applied to a wide range of task-switching paradigms, whenever both task-relevant and task-irrelevant features vary from trial to trial.

In a further analysis (not pre-registered), we analyzed performance as a function of task lag, i.e., how many trials back the task was last performed. We observed that RTs became faster with increasing task lag, consistent with the assumption that task-level inhibition slowly decays over the course of several trials. This effect was not significantly modulated by episodic interference condition, and also on a descriptive level, we did not observe any evidence for a systematic modulation of the task lag effect by episodic interference. This data pattern thus provides an empirical confirmation of the assumption that task-level inhibition slowly decays over time, a theoretical assumption that is crucial for the interpretation of N-2 task repetition costs as a marker of inhibition, but seldomly put to empirical test. To summarize, we observed evidence for both task-level inhibition and for episodic interference in our data, as indicated by the main effects of task lag and of interference condition, respectively.

### Episodic interference effects in other cognitive control measures

The current research question of whether N-2 task repetition costs reflect inhibition, as previously assumed, or whether they can be explained in terms of episodic intereference, relates to an emerging literature on the general question of how to control for episodic interference effects in cognitive-control measures. For instance, when measuring the control process of conflict adaptation, the sequential congruency effect is one of the most popular behavioral measures (Botvinick et al., 2001; Gratton et al., 1992). Here, a potential confound with episodic interference effects has long been recognized (Hommel et al., 2004; Mayr & Awh, 2009; Mayr et al., 2003; Ullsperger et al., 2005). Researchers have developed several methods of controlling for episodic interference when using the sequential congruency effect (for reviews, see e.g., Duthoo et al., 2014a, 2014b; Egner, 2007; 2017). In general, it is recommended to minimize episodic interference effects by avoiding repetitions of parts of the previous episode (e.g., repetition of stimulus and/or response across episodes), either by analysis or by design.

Apart from sequential congruency effects, other cognitive control measures potentially suffer from the same problem. Another prominent example is the negative priming effect, which originally was interpreted in terms of persisting inhibition of previously ignored stimulus features (Tipper, 1985), but could also reflect episodic interference effects (e.g., Neill, 1997; MacLeod et al., 2003; see Frings et al., 2015; Tipper, 2001, for reviews). In the domain of task switching, task-switch costs (i.e., the performance difference between task-switch trials and task-repetition trials) have been taken as indicator of the cognitive-control process of task-set reconfiguration (time for establishing a new task-set and/or time needed to overcome the previous task-set configuration; Kiesel et al., 2010; Monsell, 2003). Yet, presenting a task cue re-activates the previous episode associated with that task cue, raising the possibility that episodic effects contribute to the empirical measure of task-switch costs. One possibility of minimizing cue-triggered episodic interference effects in task switching is to introduce different cues for the same task, thereby disentangling effects of cue repetition and task repetition (see Jost et al., 2013, for review; see Altmann, 2007; Gade & Koch, 2008, for assessing N-2 task repetition costs with different cues for the same task). To conclude, disentangling the contributions of episodic interference effects and cognitive control processes is an important issue to consider in the cognitive control literature, whenever assessing cognitive control functions via sequential effects in behavioral paradigms.

## Conclusion

Inhibition of task sets and episodic interference between task episodes probably both contribute to N-2 task repetition costs in task switching. We cannot fully determine the relative contributions of the two mechanisms to N-2 task-repetition costs on the basis of the present data, but we do observe empirical markers of both task-set inhibition and episodic interference: Performance became better the more time passed (or the more intervening tasks occurred) before switching back to a previously performed task, consistent with the notion of task-level inhibition that decays slowly over time. Performance also depended on the degree of episodic interference between current and previous episode of the same task, with best performance when the two episodes fully matched, intermediate when they matched with respect to the task-relevant (but not task-irrelevant) features, and worst performance when they fully mismatched. This data pattern suggests that there is episodic interference between two episodes of the same task in a task-switching paradigm even when several seconds passed between the two episodes, and several intervening tasks lay in between.

## Data Accessibility Statements

The raw data can be downloaded from https://osf.io/qp5eh/.

Experiment 1 was pre-registered at https://aspredicted.org/iw63x.pdf.

## Additional File

The additional file for this article can be found as follows:

10.5334/joc.244.s1 Online Supplementary Material.

## Footnotes

Partial-repetition costs are usually computed as the performance difference between conditions where either no feature repeats *or all features repeat*, and conditions where some features repeat, but other do not. Here, we focus on the comparison between a condition where no feature repeats and a condition where some features repeat.

In the formal model of Altmann (2011), the degree of episodic overlap is quantified in terms of binary associations between two features; the (mis)match score is the difference between the number of binary associations that mismatch minus the number of binary associations that match.

For instance, in Grange et al. (2017), Experiment 3, stimulus mismatch condition, the numerical RT difference between the two CBA conditions was about 45 ms, with slower RTs in N-2 response repetitions than N-2 response switches. In Kowalczyk & Grange (2019), in the data set labelled “aging experiment”, the numerical difference in error rates between the CBA conditions was about 1.7% (4.4% in N-2 response repetitions versus 2.7% in N-2 response switches). In Grange (2018) and Grange et al. (2019), log-transformed RTs are pictured, making it difficult to judge whether there were RT differences between the CBA conditions. Error rates were always numerically higher in the CBA conditions with N-2 response repetitions than in the CBA conditions with N-2 response switches; the CBA conditions differed up to about 0.7% (2.5% versus 1.8% in Session 3 of the experiment reported in Grange et al., 2019). In all of these instances, the difference between the two CBA conditions was in the same order of magnitude as the overall N-2 task-repetition cost observed in those studies. Note that the difference cannot be explained by assuming residual activation of the response executed in trial N-2, because such a mechanism would imply *better* performance in N-2 response repetitions than in N-2 response switches, wheras the opposite data pattern (*worse* performance in N-2 response repetitions than switches) was observed in the CBA conditions in the above-mentioned studies.

From the description in Gade et al. (2016), it does not become entirely clear whether CBA sequences were also separated into N-2 stimulus repetitions versus switches, or whether all CBA sequences were used as a common baseline to compute N-2 task-repetition costs in N-2 stimulus repetitions and N-2 stimulus switches.

The main aim of the study by Gade et al. (2016) was to demonstrate a working-memory analogue of N-2 task-repetition costs in a working-memory paradigm. In every trial, participants had to retrieve one of several previously learned lists of items from memory, and perform mental operations on specific list features. The authors observed N-2 *list* repetition costs (in analogy to N-2 *task* repetition costs in task switching). These list repetition costs, which could be interpreted as a marker of inhibitory control, were also influenced by episodic retrieval effects: When the same list had to be retrieved in trial N-2 and N, and when also the relevant list feature repeated from N-2 to N, Gade et al. (2016) observed attenuated N-2 list repetition costs, or even N-2 list repetition benefits. Gade et al. (2016) concluded that episodic interference plays a role both in N-2 *list*-repetition costs and in N-2 *task*-repetition costs.

In the study by Schuch & Konrad (2017), N-2 task repetition costs were compared between a group of young adults and a group of children; in Schuch (2016), between younger and older adults. Because N-2 task repetition costs might differ between different age groups, we restricted our re-analysis to the young adults groups from both studies.
