# Supplementary material for: N-2 Repetition Costs in Task Switching: Task Inhibition or Interference Between Task Episodes?
Source: J Cogn. 2022 Nov 4;5(1):48. doi: 10.5334/joc.244 (PMC9635329; doi:10.5334/joc.244)
Supplement: Online Supplementary Material. [file joc-5-1-244-s1.pdf]

Online Supplementary Material

This online supplementary material belongs to the following article:
Schuch, S. & Keppler, E. (2022). N-2 repetition costs in task switching: Task inhibition or interference between task episodes? Journal of Cognition.

Overview of Online Supplementary Material:

N-X contrast

- N-X contrast: Mean number of trials per participant and condition
- Additional analysis I: N-X contrast for Experiment 1 data excluding trials with an error during the last episode of task A in CBA task sequences
- Additional analysis II: N-X contrast for Experiment 1 data excluding all task sequences with an error during the five preceding trials, and including only task sequences with up to lag 5 task repetitions
- Exploratory task-lag analysis: Descriptive data presented in Figure 5 in main text, including standard errors of mean

N-2 contrast

- N-2 contrast: N-2 task repetition costs as a function of N-2 stimulus/response transition

Diffusion model (DM) analysis

- DM analysis of the previously published data
- DM analysis of Experiment 1
- Graphical illustration of diffusion model fit

References

N-X contrast

N-X contrast: Mean number of trials per participant and condition

Table S1. Mean number of trials per participant and condition (SD and range in parentheses) included in the N-X contrast analysis of mean performance reported in the main text.

	Task Sequence	
	**ABA**	**CBA**
**Re-analysis of the data from Schuch & Koch (2017) and Schuch (2016):**		
**RT analysis**		
Episodic match of task-relevant features	53.0 (5.4) (39-62)	44.2 (5.2) (29-52)
Episodic mismatch of task-relevant and task-irrelevant features	43.6 (5.6) (29-52)	49.4 (5.2) (35-57)
**Error rate analysis**		
Episodic match of task-relevant features	55.4 (4.2) (44-63)	45.8 (4.2) (33-53)
Episodic mismatch of task-relevant and task-irrelevant features	46.7 (3.6) (37-52)	51.9 (3.8) (41-58)
**Experiment 1:**		
**RT analysis**		
Episodic match of task-relevant and task-irrelevant features	160.3 (20.1) (104-184)	92.0 (12.4) (65-110)
Episodic match of task-relevant features	257.3 (32.6) (160-302)	284.2 (38.9) (166-334)
Episodic mismatch of task-relevant and task-irrelevant features	363.0 (56.1) (189-435)	404.4 (58.8) (241-479)
**Error rate analysis**		
Episodic match of task-relevant and task-irrelevant features	164.6 (17.6) (116-185)	95.7 (9.8) (69-110)
Episodic match of task-relevant features	269.2 (26.3) (184-302)	298.5 (30.4) (207-335)
Episodic mismatch of task-relevant and task-irrelevant features	386.5 (40.1) (264-437)	430.7 (42.6) (306-480)

Additional analysis I: N-X contrast for Experiment 1 data excluding trials with an error during the last episode of task A in CBA task sequences

In the pre-registration of Experiment 1, data filtering was specified in the following way: “Data filtering for analysis of mean performance: The first two trials from each experimental block, the two trials following an error, and outliers (RT deviating more than three SDs from a participants’ overall mean RT) will be excluded. For RT analysis, error trials will be excluded as well.”

However, as pointed out by a Reviewer, excluding the two trials following each error means that in ABA task sequences, the performance of task A in trial N-2 was always error-free, whereas in CBA sequences, the last performance of task A (in trial N-3 or further back) could have been associated with an error. In order to exclude this potential bias, we re-ran the analysis reported in the main text (and presented in Figure 4 in the main study), and additionally excluded all CBA sequences in which participants had made an error during the last episode of task A (in trial N-3 or further back). The results were virtually identical to those reported in the main text, suggesting that including or excluding those error trials did not induce any systematic bias.

In detail, the ANOVA on RTs disclosed a significant main effect of N-2 Task Transition $F(1, 39) = 27.98$, $p < .001$, $\eta^2_p = .42$, indicating N-2 task repetition costs, and a significant main effect of Episodic Match Condition, $F(2, 78) = 19.97$, $p < .001$, $\eta^2_p = .34$, indicating slower RTs with increasing mismatching features. The two-way interaction was not significant, $F(2, 78) = 1.56$, $p = .22$, $\varepsilon = 0.69$, indicating that N-2 task repetition costs did not statistically differ between the three episodic conditions. When analyzed separately for the different episodic conditions, N-2 task repetition costs were significant in the mismatch condition (mean = 37 ms, SEM = 5 ms, $t(39) = 6.91$, $p < .001$, $d = 1.11$) and in the condition of episodic match of task-relevant features (mean = 32 ms, SEM = 7 ms, $t(39) = 4.32$, $p <$

.001, *d* = 0.69), but not in the full episodic match condition (mean = 18 ms, SEM = 11 ms, *t*(39) = 1.55, *p* = .13).

The corresponding ANOVA on error rates revealed a significant main effect of N-2 Task Transition, *F*(1, 39) = 4.76, *p* = .035, η^2_p = .11, indicating a N-2 task repetition *benefit* across all conditions. There was also a significant main effect of Episodic Match Condition, *F*(2, 78) = 31.18, *p* < .001, η^2_p = .44, ε = 0.72, indicating that error rates became higher with increasing mismatching features. The two-way interaction was not significant, *F*(2, 78) = 1.65, *p* = .199. When analyzed separately for the different episodic conditions, N-2 task repetition costs were not significant in the mismatch condition (mean N-2 task repetition cost = 0.02%, SEM = 0.35%, *t*(39) < 1), and neither in the condition of episodic match of task-relevant features (mean = -0.34%, SEM = 0.47%, *t*(39) < 1); in the condition of full episodic matches, a significant negative N-2 repetition cost (i.e., N-2 repetition benefit) was obtained (mean = -1.06%, SEM = 0.40%, *t*(39) = 2.65, *p* = .012, *d* = 0.42).

Additional analysis II: N-X contrast for Experiment 1 data excluding all task sequences with an error during the five preceding trials, and including only task sequences with up to lag 5 task repetitions.

Another consequence of our pre-registered data filtering procedure is that intermediate errors may occur between the last and current episode of task A in CBA task sequences; e.g., in a ABCBA task sequence, while performing both episodes of task A correctly, the participant might have committed an error while performing task B in trial N-3. In order to rule out potential influences of such intermediate errors, we ran a second additional analysis. We excluded *five* trials after each error (instead of only *two* trials after error as in the main analysis). Hence, in this analysis we only analyzed trials that were preceded by at least five correct trials. Moreover, we only included CBA task sequences with N-3, N-4, and N-5 task repetitions (i.e., only CBA task sequences of the kinds ACBA, ABCBA, and ACBCBA). Note that in this way, still about 87% of all CBA task sequences were included; for distribution of task lags, see main text). Table S2 shows the mean number of trials per participant and condition for this analysis.

The results were similar to the N-X contrast analysis reported in the main text, except that N-2 task-repetition costs in RT were now significant event in the condition of full episodic matches. In detail, the ANOVA on RTs revealed a significant main effect of N-2 Task Transition $F(1, 39) = 31.23$, $p < .001$, $\eta^2_p = .45$, indicating N-2 task repetition costs, and a significant main effect of Episodic Match Condition, $F(2, 78) = 15.84$, $p < .001$, $\eta^2_p = .29$, $\varepsilon = 0.86$, indicating slower RTs with increasing mismatching features. The two-way interaction was not significant, $F(2, 78) < 1$, $\varepsilon = 0.70$, indicating that N-2 task repetition costs did not statistically differ between the three episodic conditions. When analyzed separately for the different episodic conditions, N-2 task repetition costs were significant in the mismatch condition (mean = 36 ms, SEM = 6 ms, $t(39) = 6.08$, $p < .001$, $d = 0.97$) and the condition of episodic match of task-relevant features (mean = 27 ms, SEM = 7 ms, $t(39) = 3.70$, $p < .001$, d

= 0.59), and also in the full episodic match condition (mean = 30 ms, SEM = 12 ms, $t(39) = 2.44$, $p < .02$, $d = 0.39$).

In the ANOVA on error rates, the main effect of N-2 Task Transition was not significant, $F(1, 39) = 3.78$, $p = .06$, $\eta^2_p = .09$, indicating a N-2 task repetition *benefit* across all conditions. There was also a significant main effect of Episodic Match Condition, $F(2, 78) = 29.21$, $p < .001$, $\eta^2_p = .43$, $\varepsilon = 0.72$, indicating that error rates became higher with increasing mismatching features. The two-way interaction was not significant, $F(2, 78) = 1.00$. When analyzed separately for the different episodic conditions, N-2 task repetition costs were not significant in the mismatch condition (mean N-2 task repetition cost = -0.39%, SEM = 0.34%, $t(39) = 1.15$), and neither in the condition of episodic match of task-relevant features (mean = -0.23%, SEM = 0.51%, $t(39) < 1$); in the condition of full episodic matches, a significant negative N-2 repetition cost (i.e., N-2 repetition benefit) was obtained (mean = -1.00%, SEM = 0.42%, $t(39) = 2.39$, $p = .022$, $d = 0.38$).

Table S2. Mean number of trials per participant and condition (SD and range in parentheses) for the N-X contrast when including only trials that were preceded by at least five correct trials, and including only task sequences with N-5 or sooner task repetitions.

	Task Sequence	
	**ABA**	**CBA**
**Experiment 1:**		
**RT analysis**		
Episodic match of task-relevant and task-irrelevant features	137.9 (32.8) (57-179)	63.1 (15.0) (30-85)
Episodic match of task-relevant features	220.4 (53.1) (87-297)	220.8 (55.0) (83-297)
Episodic mismatch of task-relevant and task-irrelevant features	313.3 (79.9) (108-431)	305.4 (79.5) (94-410)
**Error rate analysis**		
Episodic match of task-relevant and task-irrelevant features	141.3 (31.0) (63-180)	65.2 (13.9) (33-85)
Episodic match of task-relevant features	229.9 (49.1) (102-297)	230.6 (50.4) (98-297)
Episodic mismatch of task-relevant and task-irrelevant features	330.9 (70.4) (142-433)	323.6 (69.7) (123-411)

Exploratory task-lag analysis: Descriptive data presented in Figure 5 in main text, including standard errors of mean.

Table S3. Mean RTs and Error Rates (standard error of mean in parentheses), as a function of Task Lag and Episodic Match Condition.

	**Task Sequence**		
	**ABA (lag=2)**	**ACBA (lag=3)**	**A…CBA (lag>3)**
**Re-analysis of the data from Schuch & Koch (2017) and Schuch (2016):**			
**RT analysis**			
Episodic match of task-relevant features	925 (31)	907 (31)	868 (31)
Episodic mismatch of task-relevant and task-irrelevant features	933 (32)	909 (34)	875 (34)
**Error rate analysis**			
Episodic match of task-relevant features	4.6% (0.5%)	4.8% (0.8%)	2.7% (0.5%)
Episodic mismatch of task-relevant and task-irrelevant features	6.9% (0.8%)	5.4% (0.7%)	4.7% (0.6%)

	**ABA (lag=2)**	**ACBA (lag=3)**	**ABCBA (lag=4)**	**ACBCBA (lag=5)**	**A…CBCBA (lag>5)**
**Experiment 1:**					
**RT analysis**					
Episodic match of task-relevant and task-irrelevant features	882 (45)	896 (51)	833 (44)	846 (52)	856 (52)
Episodic match of task-relevant features	919 (48)	889 (47)	881 (47)	908 (55)	868 (48)
Episodic mismatch of task-relevant and task-irrelevant features	924 (47)	905 (44)	880 (46)	860 (41)	861 (44)
**Error rate analysis**					
Episodic match of task-relevant and task-irrelevant features	2.8% (0.3%)	3.6% (0.5%)	4.9% (0.8%)	4.0% (0.8%)	4.9% (1.0%)
Episodic match of task-relevant features	4.7% (0.5%)	5.1% (0.7%)	4.7% (0.6%)	5.4% (0.7%)	5.4% (0.8%)
Episodic mismatch of task-relevant and task-irrelevant features	6.6% (0.8%)	6.5% (0.7%)	6.4% (0.7%)	7.1% (0.9%)	6.5% (0.9%)

N-2 contrast

N-2 contrast: N-2 task repetition costs as a function of N-2 stimulus/response transition

We also analyzed the data using the N-2 contrast, similar to the contrast used in the work by Mayr (2002) and Grange and colleagues. For the re-analysis of the previously published data, a 2x2 within-subject design was applied with the independent variables N-2 Task Transition (ABA, CBA) and N-2 Response Transition (N-2 response repetition vs N-2 response switch). For Experiment 1, a 2x3 within-subject design was applied with the independent variables N-2 Task Transition (ABA, CBA) and N-2 Stimulus/Response Transition (N-2 repetition of stimulus and response, N-2 repetition of response only, N-2 switch of stimulus and response).

Re-analysis of the previously published data. The descriptive data are presented in Figure S1. The ANOVA on mean reaction times (RTs) revealed a main effect of Task Transition, $F(1,55) = 33.3$, $p < .01$, $\eta^2_p = .38$, indicating the standard finding of N-2 task repetition costs. There was no main effect of Response Transition, $F(1,55) = 2.53$, $p = .12$. Importantly, the two-way interaction was not significant, $F(1,55) < 1$, indicating that N-2 task repetition costs did not statistically differ between N-2 response repetitions and switches. When analyzed separately, N-2 task repetition costs were 47 ms in N-2 response repetitions (standard error of mean [sem] 10 ms), $t(55) = 4.62$, $p < .01$, and 39 ms in N-2 response switches (sem 12 ms), $t(55) = 3.28$, $p < .01$.

In Error Rates, the respective ANOVA revealed a main effect of Task Transition, $F(1,55) = 10.30$, $p < .01$, $\eta^2_p = .16$, indicating N-2 task repetition costs, and a main effect of Response Transition, $F(1,55) = 11.53$, $p < .01$, $\eta^2_p = .16$, indicating lower error rates in N-2 response repetitions than in N-2 response switches. The interaction was marginally significant, $F(1,55) = 2.97$, $p = .09$, $\eta^2_p = .05$, indicating a trend for smaller N-2 task repetition costs in N-2 response repetitions (costs of 0.7%, sem 0.5%, $t(55) = 1.31$, n.s.) than in N-2 response switches (costs of 2.2%, sem 0.7%, $t(55) = 2.97$, $p < .01$).

Experiment 1. There were a small number of CBA trials with N-2 stimulus repetition but N-2 response switch; these trials were excluded from the N-2 contrast analysis with N-2 Transition as independent variable because they could not be classified into any of the three levels of this independent variable.

The descriptive data are presented in Figure S2. The ANOVA on mean RTs revealed a significant main effect of N-2 Task Transition, suggesting that N-2 repetition costs are present when averaged across all N-2 Stimulus/Response Transition conditions, $F(1, 39) = 41.46$, $p < .001$, $\eta^2_p = .52$. Moreover, the main effect of N-2 stimulus/response transition was significant, $F(2, 78) = 18.95$, $p < .001$, $\eta^2_p = .33$, $\varepsilon = 0.61$, indicating that the RTs significantly differ between the three N-2 Stimulus/Response Transition conditions. Critically, the two-way interaction between N-2 Stimulus/Response Transition and N-2 Task Transition was not significant, $F(2, 78) = 1.89$, $p = .157$, suggesting that N-2 repetition costs do not differ between the N-2 Stimulus/Response Transition conditions. When analyzed separately, N-2 repetition costs were 29 ms when stimulus and response repeated from N-2 to N (SEM = 11 ms, $t(39) = 2.79$, $p = .008$, $d = .44$); 25 ms when only the response repeated from N-2 to N (SEM = 8 ms, $t(39) = 3.09$, $p = .004$, $d = 0.49$); and 47 ms when both stimulus and response switched from N-2 to N (SEM = 7 ms, $t(39) = 6.84$, $p < .001$, $d = 1.08$).

The ANOVA on error rates revealed a different pattern of results: Here, a significant two-way interaction between N-2 Task Transition and N-2 Stimulus/Response Transition was observed, $F(2, 78) = 9.80$, $p < .001$, $\eta^2_p = .20$, $\varepsilon = 0.83$, as well as significant main effects of N-2 Task Transition, $F(1, 39) = 5.62$, $p = .023$, $\eta^2_p = .13$, and of N-2 Stimulus/Response Transition, $F(2, 78) = 20.94$, $p < .001$, $\eta^2_p = .20$. Paired *t*-tests revealed that there were significant differences between ABA and CBA task sequences in all three N-2 Stimulus/Response Transition conditions, but in different directions: When both stimulus and response switched from N-2 to N, N-2 repetition costs of 1.45% were obtained, $t(39) = 3.64$, $p = .001$, $d = 0.57$. However, when the response repeated from N-2 to N, there was a significant

N-2 repetition *benefit* of 1.60%, $t(39) = 3.82$, $p < .001$, d = 0.60; when both stimulus and response repeated from N-2 to N, there was also a N-2 repetition *benefit* of 1.19%, $t(39) = 2.07$, $p = .045$, $d = 0.33$.

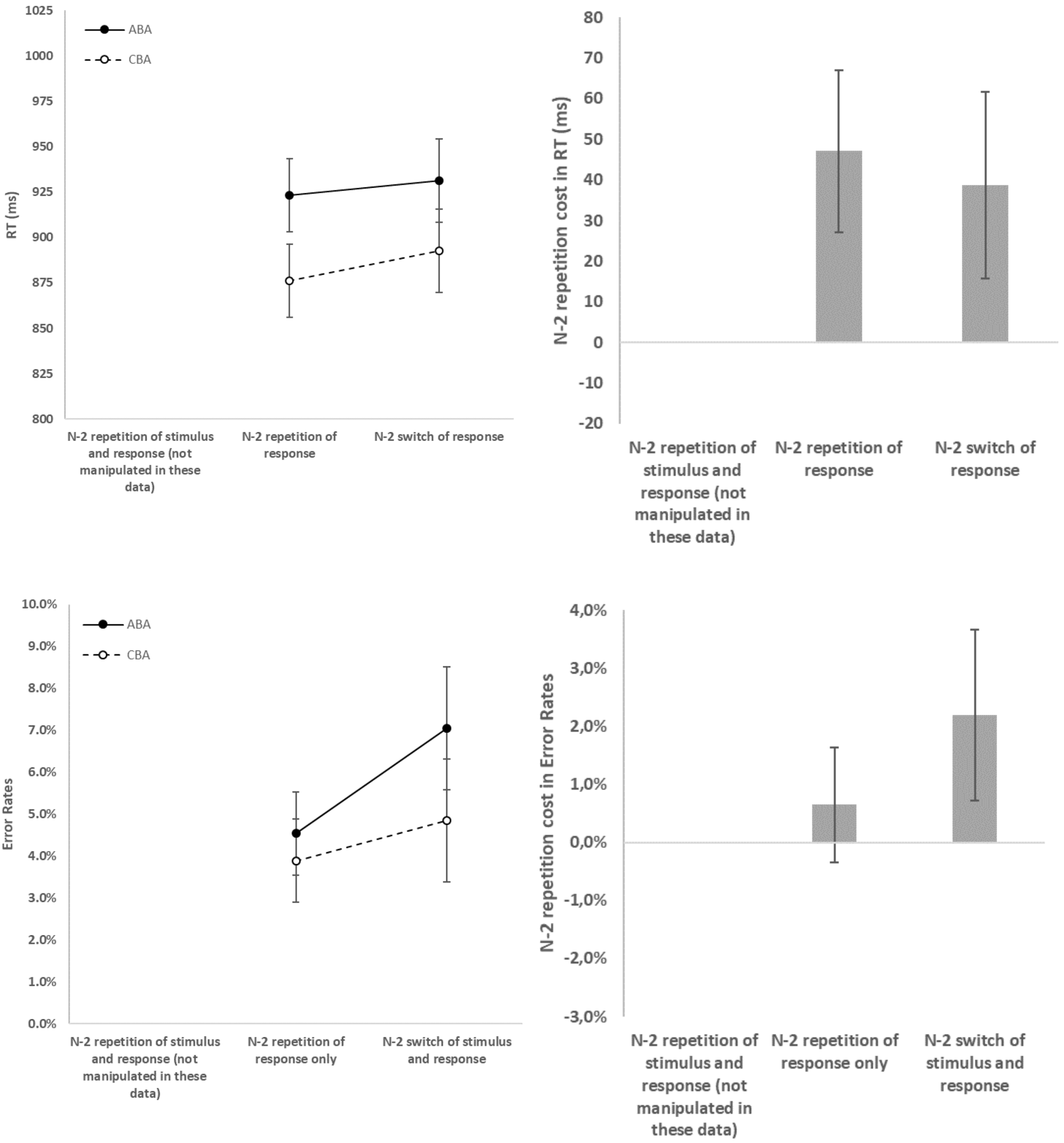

Figure S1. Re-analysis of the young adults' groups from Schuch & Konrad (2017) and Schuch (2016). N=56. Left side: Mean RT (upper row) and mean Error Rate (lower row) as a function of N-2 Task Transition (N-2 Task Repetition [ABA] versus N-2 Task Switch [CBA]) and N-2 Response Transition (N-2 repetition of response, N-2 switch of response). Right side: N-2 task-repetition costs as a function of N-2 Response Transition in RTs and Error Rates. Error bars represent the 95% confidence interval (CI) of the ABA-CBA difference per N-2 Response Transition Condition (Pfister & Janczyk, 2013) in both left and right panel; in the left panel, the difference between mean ABA and mean CBA corresponds to a significant paired-samples t-test if one mean is not included in the CI around the other mean. Note: The data points in the ABA condition are identical to those in Figure 3 in main text; only the data points in the CBA condition differ between the analyses.

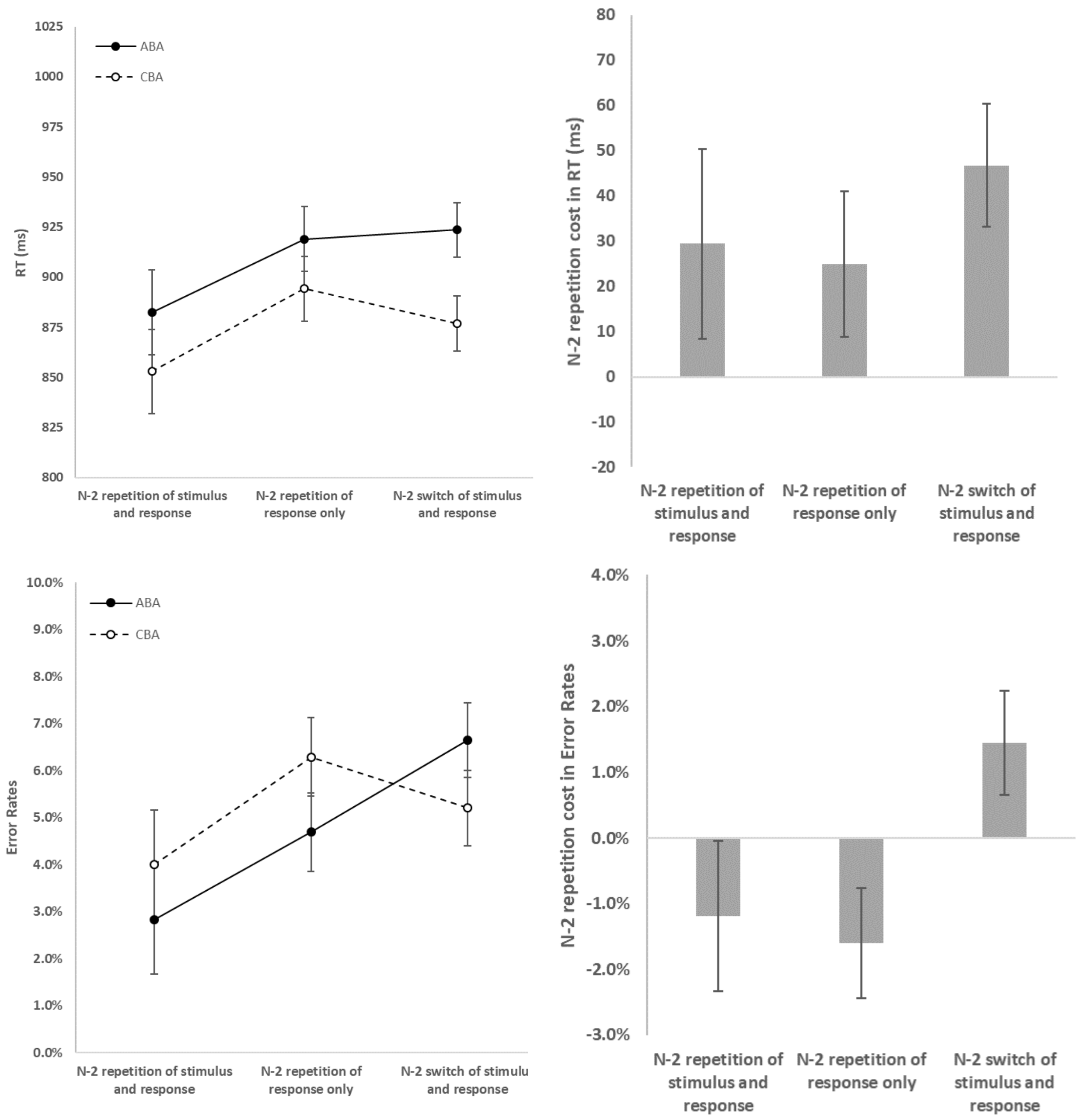

Figure S2. Experiment 1. N=40. Left side: Mean RT (upper row) and mean Error Rate (lower row) as a function of N-2 Task Transition (N-2 Task Repetition [ABA] versus N-2 Task Switch [CBA]) and N-2 Stimulus/Response Transition (N-2 repetition of stimulus and response, N-2 repetition of response only, N-2 switch of stimulus and response). Right side: N-2 task-repetition costs as a function of N-2 Response Transition in RTs and Error Rates. Error bars represent the 95% confidence interval of the ABA-CBA difference per N-2 Stimulus/Response Condition (Pfister & Janczyk, 2013). Note: The data points in the ABA condition are identical to those in Figure 4 in main text; only the data points in the CBA condition differ between the analyses.

Diffusion model analysis

We also performed diffusion modeling of our data, in order to further investigate the potential contributions of task inhibition and episodic interference to N-2 task-repetition costs. In general, the diffusion model assumes that reaction times (RT) in speeded choice RT tasks can be fractionated into a decisional and a non-decisional phase. During the decisional phase, evidence for the possible response alternatives is accumulated. As soon as the evidence has reached a certain threshold, the respective response alternative is selected and executed. In the simplest version of the diffusion model, the decisional phase is characterized by the average rate of evidence accumulation (drift rate parameter) and the amount of evidence that needs to be accumulated (boundary separation parameter); the non-decisional component subsumes all cognitive processes outside the decision process and is characterized by one further parameter (non-decision time parameter).

Kowalczyk & Grange (2019) applied diffusion modeling to investigate the contributions of task inhibition and episodic retrieval to N-2 task repetition costs using Mayr's (2002) spatial transformation tasks paradigm. Across four experiments, Kowalczyk & Grange (2019) found that N-2 task repetition costs were reflected in drift rate, with smaller drift rate in ABA than CBA, consistent with earlier diffusion modeling findings from Schuch and colleagues (Schuch, 2016; Schuch & Grange, 2019; Schuch & Konrad, 2017; see also Moretti, Koch, Steinhauser, & Schuch, 2021). Importantly, in Kowalczyk & Grange's (2019) analyses, the N-2 repetition cost in drift rate was modulated by episodic interference: it was only significant in N-2 response switches, but not in N-2 response repetitions. This led those authors to conclude that the effect in drift rate reflects interference due to episodic mismatch, rather than persisting task inhibition. Here, we further explore whether the N-2 task repetition cost drift rate is affected by the degree of episodic interference also in a task switching paradigm with categorization tasks.

Beyond drift rate, also the boundary separation parameter was of interest to us. In Experiment 1, we had unexpectedly oberserved N-2 task repetition *costs* in RTs with simultaneous N-2 task repetition *benefits* in error rates. This could point to a shift of speed-accuracy tradeoff between ABA and CBA conditions. Numerically, the tradeoff between N-2 task repetition *costs* in RTs and *benefits* in error rates was most pronounced in the condition of full episodic matches. Interestingly, the pattern of N-2 repetition cost in RT with simultaneous N-2 repetition benefit in error data in the full episodic match condition has been observed previously. For instance, it was observed in the studies by Grange et al. (2019), Grange (2018, condition with short response-cue interval), and Grange et al. (2017, Experiment 2; episodic matches were defined as N-2 response repetitions in these studies). Grange et al. (2019, p. 67) discuss the possibility that this data pattern might indicate a strategic upregulation of response caution in the ABA condition of full episodic match. The present diffusion model analysis allows us to further explore this issue.

Method of diffusion model analysis

Data filtering. Data filtering was the same as for the analysis of mean performance, except that RT oultiers were defined according to the procedure recommended by Schmiedek et al. (2007): Trials with RTs faster than 200 ms were excluded, as well as trials with RTs larger than four standard deviations above each participant's mean per experimental condition were excluded; this criterion was applied to the remaining trials repeatedly until there were no further outliers. In the data sets of Schuch & Konrad (2017) and Schuch (2016), the four participants that were excluded from diffusion model analyis in those studies were excluded here as well, leaving N=50 participants for the present diffusion-model analysis. (These four participants were excluded because they showed N-2 repetition costs that were larger than two times the interquartile range in those studies; the present diffusion modeling results were similar when all participants [N=56] were included.) For diffusion modeling of Experiment 1, all participants (N=40) were included.

Model parameterisation and fit procedure. These were the same as in Schuch and Konrad (2017) and Schuch (2016), except that maximum likelihood (ML) was chosen as optimization criterion, because trial numbers were relatively low (Lerche, Voss, & Nagler, 2017). Model fit was inspected graphically with quantile-quantile plots, where empirical data are plotted against data predicted by the model (see Figures S3 and S4). The software fast-dm (Voss & Voss, 2007; Voss, Voss, & Lerche, 2015) was used to estimate the three parameters drift rate (v), threshold separation (a), and non-decision time (t0) separately for each individual and each condition. In order to improve model fit, variability of non-decision time (st0) was also free to vary across individuals and conditions. The starting point bias was set to 0.5a (i.e., in the middle between the two thresholds); all other parameters implemented in fast-dm were set to 0. The upper and lower thresholds corresponded to correct and error responses, respectively. Separate ANOVAs were computed on the mean parameter values of boundary separation, drift rate, and non-decision time.

Design. The independent variables were the same as for the N-X contrast (i.e., N-2 Task Transition and Episodic Match Condition); the dependent variables were the three main diffusion model parameters drift rate, boundary separation, and non-decision time.

Results of diffusion model analysis

The descriptive diffusion modeling results of the re-analyzed data and of Experiment 1 are reported in Table S4.

N-X contrast in re-analyzed data. For drift rate, the 2x2 ANOVA with the independent variables N-2 Task Transition and Episodic Match Condition revealed a main effect of N-2 Task Transition, $F(1,49) = 17.25$, $p < .01$, $\eta^2_p = .26$, indicating overall lower drift rata in ABA than CBA task sequences. There was no main effect of Episodic Match Condition, $F(1,49) = 1.50$, and no significant interaction, $F(1,49) < 1$. When tested separately, N-2 task repetition costs were significant both in episodic matches of task-relevant features

(mean = -0.16, sem = 0.06; $t(49) = 2.68$, $p = .01$), and in episodic mismatches (mean -0.24, sem 0.07; $t(49) = 3.17$, $p < .01$).

For boundary separation, the respective ANOVA yielded no main effect of N-2 Task Transition, and no interaction, $Fs(1,49) < 1$. There was a main effect of Episodic Match Condition, $F(1,49) = 8.51$, $p < .01$, $\eta^2_p = .15$, with higher boundary separation in episodic matches than mismatches. The ANOVA on non-decision time did not reveal any main effect of N-2 Task Transition, and no interaction, $Fs(1,49) < 1$. There was a main effect of Episodic Match Condition, $F(1,49) = 6.89$, $p = .01$, $\eta^2_p = .12$, indicating shorter non-decision time in episodic matches than mismatches.

N-X contrast in Experiment 1. For drift rate, the 2x3 ANOVA with the independent variables N-2 Task Transition (ABA, CBA) and Episodic Match Condition (Full Match, Match of task-relevant features, Mismatch) revealed a main effect of Episodic Match Condition, $F(2, 78) = 40.69$, $p < .01$, $\eta^2_p = .51$, $\varepsilon = 0.82$, indicating that drift rate became smaller with increasing episodic mismatch. There was no main effect of Task Transition, and no interaction, $Fs < 1.1$.

The respective ANOVA on boundary separation revealed a main effect of Episodic Match Condition, $F(2, 78) = 11.59$, $p < .01$, $\eta^2_p = .23$, $\varepsilon = 0.82$, indicating that boundary separation became smaller with increasing episodic mismatch. There was also a main effect of Task Transition, $F(1, 39) = 13.50$, $p < .01$, $\eta^2_p = .26$, indicating larger boundary with ABA than CBA, and no significant interaction, $F(2, 78) = 2.67$, $p = .08$, $\eta^2_p = .06$, $\varepsilon = 0.87$.

The respective ANOVA on non-decision time also yielded a main effect of Episodic Match Condition, $F(2, 78) = 12.95$, $p < .01$, $\eta^2_p = .25$, $\varepsilon = 0.71$, indicating increasing non-decision time with increasing episodic mismatch. There was no main effect of Task Transition, $F < 1$, and no interaction, $F(2, 78) = 2.18$, $p = .12$, $\eta^2_p = .05$.

Table S4. Diffusion modeling results. Mean parameter values per experimental condition (standard errors in parentheses).

	Task Sequence	
	**ABA**	**CBA**
**Re-analysis of the data from Schuch & Koch (2017) and Schuch (2016):**		
Drift rate parameter		
Episodic match of task-relevant features	1.768 (0.071)	1.926 (0.074)
Episodic mismatch	1.656 (0.073)	1.895 (0.071)
Boundary separation parameter		
Episodic match of task-relevant features	1.905 (0.072)	1.901 (0.075)
Episodic mismatch	1.757 (0.069)	1.776 (0.085)
Non-decision time parameter		
Episodic match of task-relevant features	0.371 (0.017)	0.368 (0.018)
Episodic mismatch	0.405 (0.021)	0.396 (0.019)
**Experiment 1:**		
Drift rate parameter		
Full episodic match	1.850 (0.075)	1.824 (0.087)
Episodic match of task-relevant features	1.686 (0.075)	1.714 (0.078)
Episodic mismatch	1.588 (0.077)	1.621 (0.075)
Boundary separation parameter		
Full episodic match	2.115 (0.104)	1.970 (0.111)
Episodic match of task-relevant features	2.001 (0.089)	1.952 (0.097)
Episodic mismatch	1.941 (0.091)	1.879 (0.091)
Non-decision time parameter		
Full episodic match	0.272 (0.018)	0.284 (0.013)
Episodic match of task-relevant features	0.308 (0.018)	0.297 (0.013)
Episodic mismatch	0.321 (0.015)	0.314 (0.012)

Discussion of diffusion model analysis

When re-analyzing the data from Schuch & Konrad (2017) and Schuch (2016) separately for episodic matches of task-relevant features (stimulus category and response) and for episodic mismatches, we did not observe any differences in N-2 repetition costs between these two episodic conditions. In both episodic conditions, N-2 task repetition costs were observed in drift rate, with lower drift rate in ABA than CBA task sequences. Hence, the diffusion modeling results did not reveal any evidence for a modulation of N-2 repetition costs in drift rate by episodic interference in the young adults' groups of Schuch & Konrad (2017) and Schuch (2016). At the same time, there were differences between the episodic interference conditions, as indicated by overall higher boundary separation and shorter non-decision time when the task-relevant features matched between the task episodes than when they did not match. This raises the possibility that participants strategically adjusted response caution, with more cautious responding in episodic matches than mismatches.

In Experiment 1, we unexpectedly did not observe the previously established effect of lower drift rate in ABA than CBA task sequences, which has been reported several times in the literature (Kowalczyk & Grange, 2019; Moretti, Koch, Steinhauser, & Schuch, 2021; Schuch, 2016; Schuch & Grange, 2019; Schuch & Konrad, 2017). Numerically, the ABA drift rate was smaller than the CBA drift rate (at least in the conditions of episodic mismatch and episodic match of task-relevant features), but there was no statistically significant effect. Given we did not observe this standard effect, it is difficult to draw conclusion about the contributions of inhibition versus episodic interference to N-2 task repetition costs, both of which would be expected to be reflected in drift rate.

Why did we not replicate the previously established drift rate effect in Experiment 1? One important difference between this Experiment and the earlier experiments from Schuch (2016) and Schuch & Konrad (2017) is the overall length of the experiment: Here, participants completed 1,920 trials, as opposed to only 240 trials in those previous studies.

That is, the present experiment comprised about eight times as many trials as the previous studies; hence, practice effects could have played a more important role here. There is some literature on the influence of practice on N-2 repetition costs; for instance, Grange and Juvina (2015), Grange et al. (2019), and Scheil (2016) all reported that N-2 repetition costs in mean RT decreased with increasing practice. However, diffusion modeling was not applied in those studies, so we do not know to date whether the N-2 repetition cost in drift rate mainly occurs when switching between relatively newly instructed tasks, and becomes attenuated with increasing practice.

While we did not find the expected N-2 repetition cost in drift rate, we did observe N-2 repetition costs in boundary separation: Participants responded more cautiously in ABA than in CBA trials (see also Kowalczyk & Grange, 2019), and this effect tended to be more pronounced with increasing episodic match. The boundary separation parameter is often thought to be under the strategic control of the participants (e.g., Hedge et al., 2019; Voss et al., 2013). Possibly, the extended length of the present experiment led participants to change their strategy of performing the task over the course of the experiment. It is conceivable that with increasing practice, participants developed a "randomness heuristic" about the random task sequence (see Schuch & Dignath, 2020), and expected that all three tasks would occur in a row (i.e. CBA) before switching back to a previously performed task (i.e., ABA). This could lead to an upregulation of response caution in the case of switching back to the N-2 task (i.e., in ABA). In a similar vein, Grange et al. (2019, p. 67) discuss the possibility of "some form of strategic change of response thresholds during episodic matches, leading to prolonged RTs and improved accuracy." The pattern of N-2 repetition *cost* in mean RT with simultaneous N-2 repetition *benefit* in mean error data thus indeed seems to reflect a shift in response caution, as evidenced by the effect in the diffusion-model boundary separation parameter.

Beyond N-2 repetition costs, some other aspects of the diffusion model analysis are noteworthy: We observed that all three main diffusion model parameters (drift rate, boundary

separation, and non-decision time) depended on the episodic interference condition. For all three, we found a roughly linear relationship with episodic interference condition: drift rate decreased with increasing episodic interference, boundary separation also decreased with increasing episodic interference, and non-decision time increased with increasing episodic interference. That is, processing becomes noisier, response caution diminishes, and non-decision time increases, with increasing episodic interference.

Graphical illustration of diffusion model fit

Figure S3. Graphical illustration of diffusion-model fit of the re-analysis of the data from Schuch & Konrad (2017) and Schuch (2016); N-X contrast.Empirical values of error proportion and of first, second, and third quartile of RT distribution of correct responses per participant and condition are plotted against the respective values predicted by the model. Filled dots indicate ABA condition, unfilled dots indicate CBA condition. Values on the diagonal would indicate perfect model fit. a) Episodic matches of task-relevant features. b) Episodic mismatches of task-relevant and task-irrelevant features.

a) Episodic Match of task-relevant features

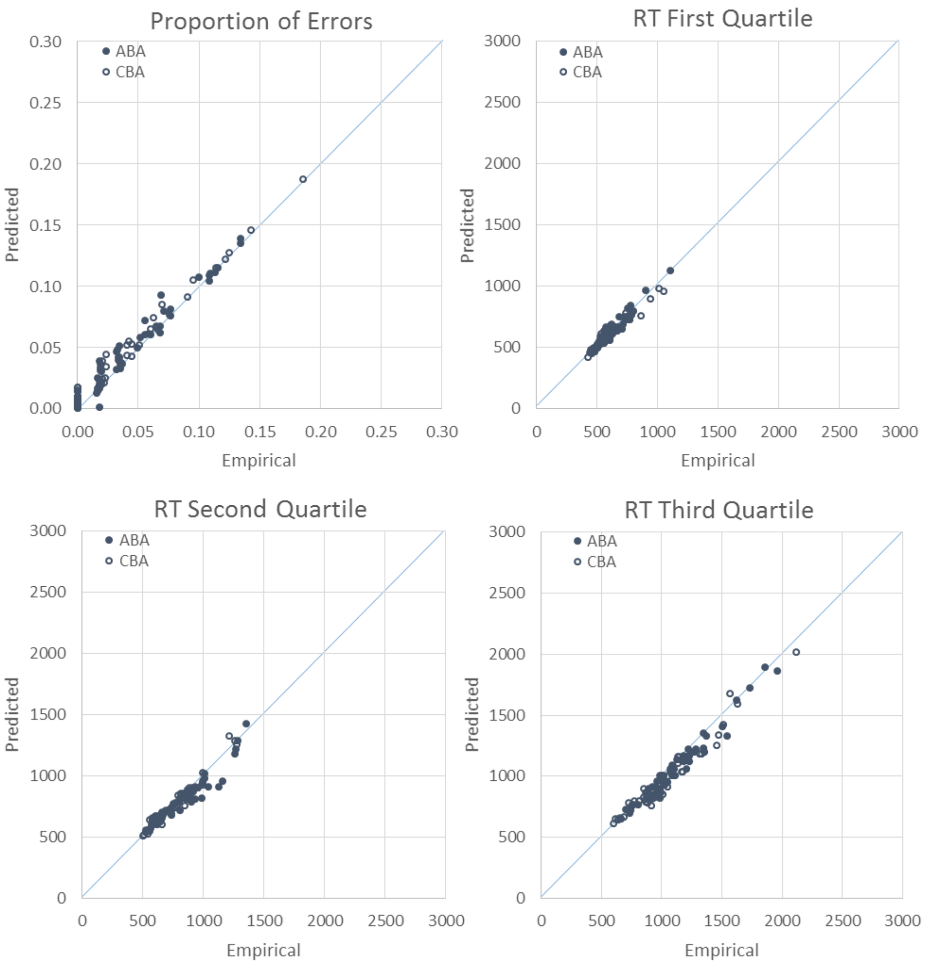

b) Episodic Mismatch of task-relevant and task-irrelevant features

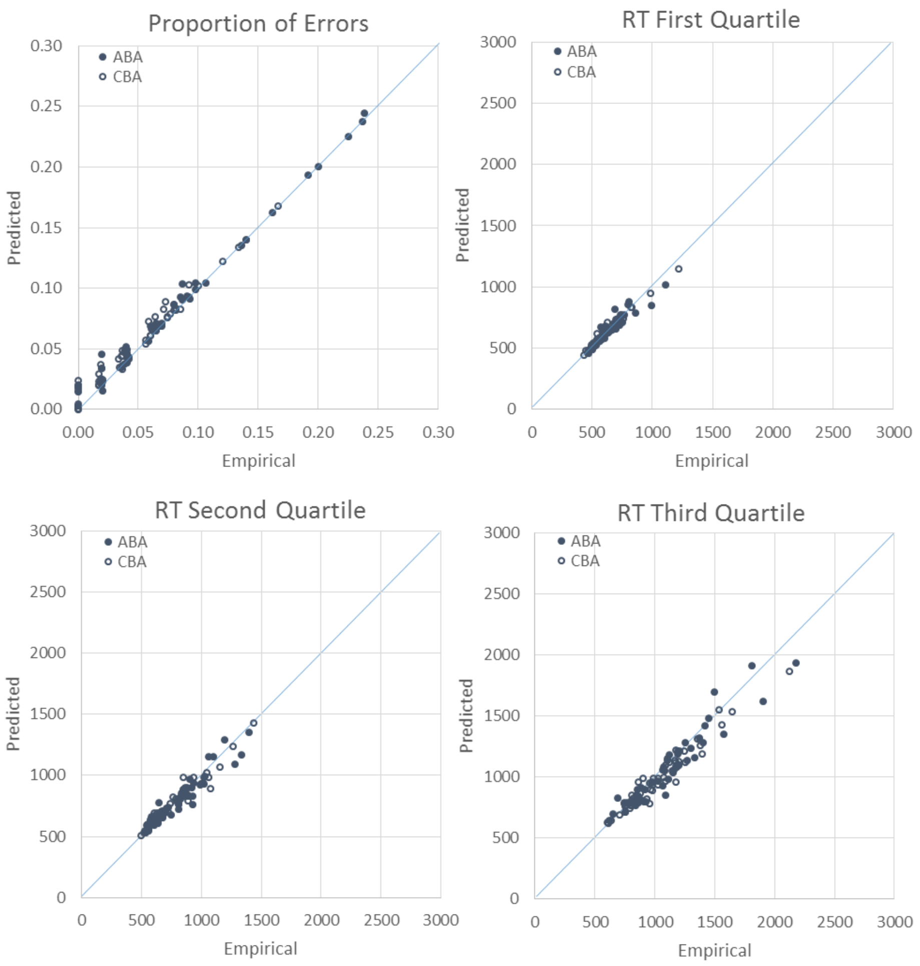

Figure S4. Graphical illustration of diffusion-model fit of Experiment 1, N-X contrast. Empirical values of error proportion and of first, second, and third quartile of RT distribution of correct responses per participant and condition are plotted against the respective values predicted by the model. Values on the diagonal would indicate perfect model fit.

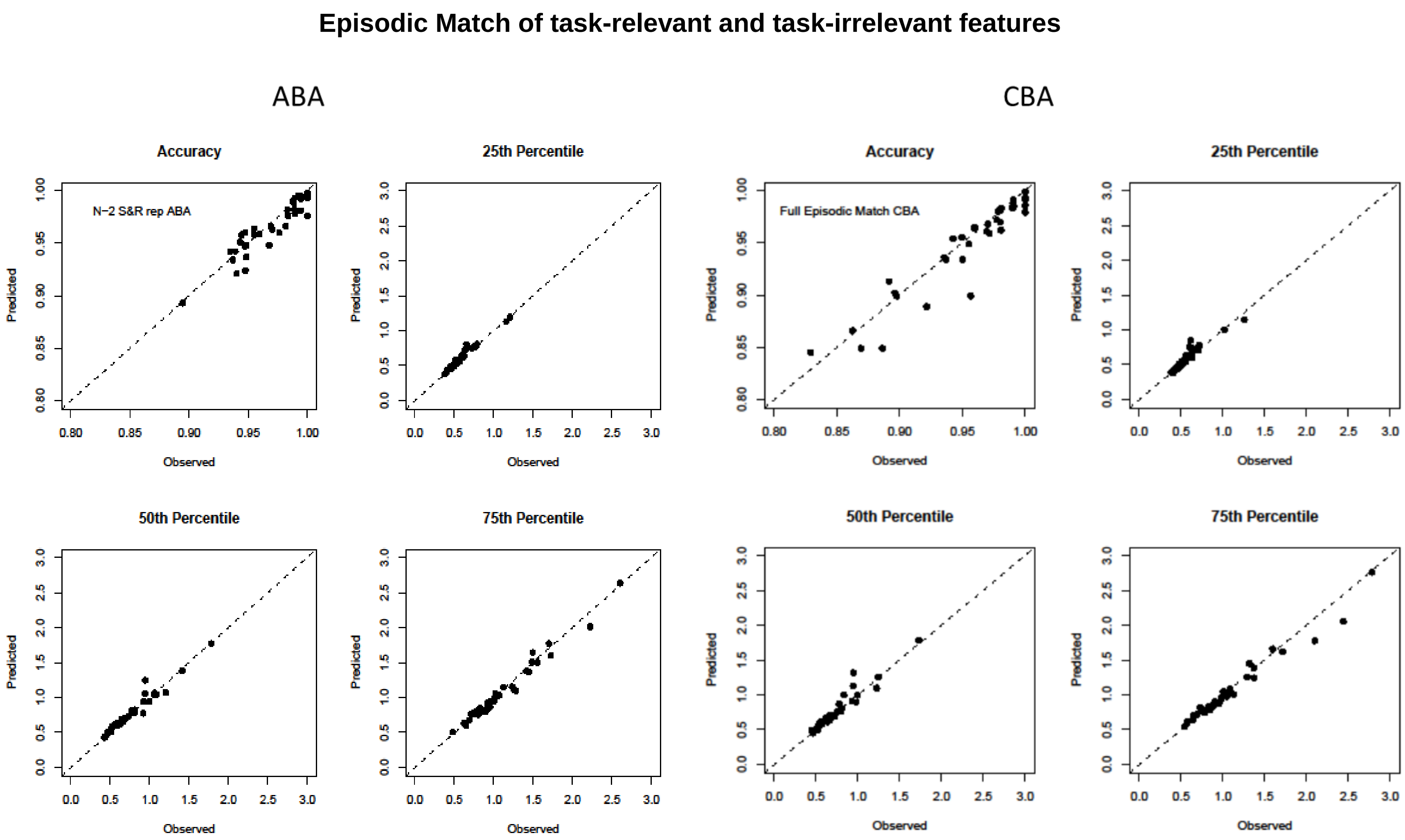

Figure S4 (continued).

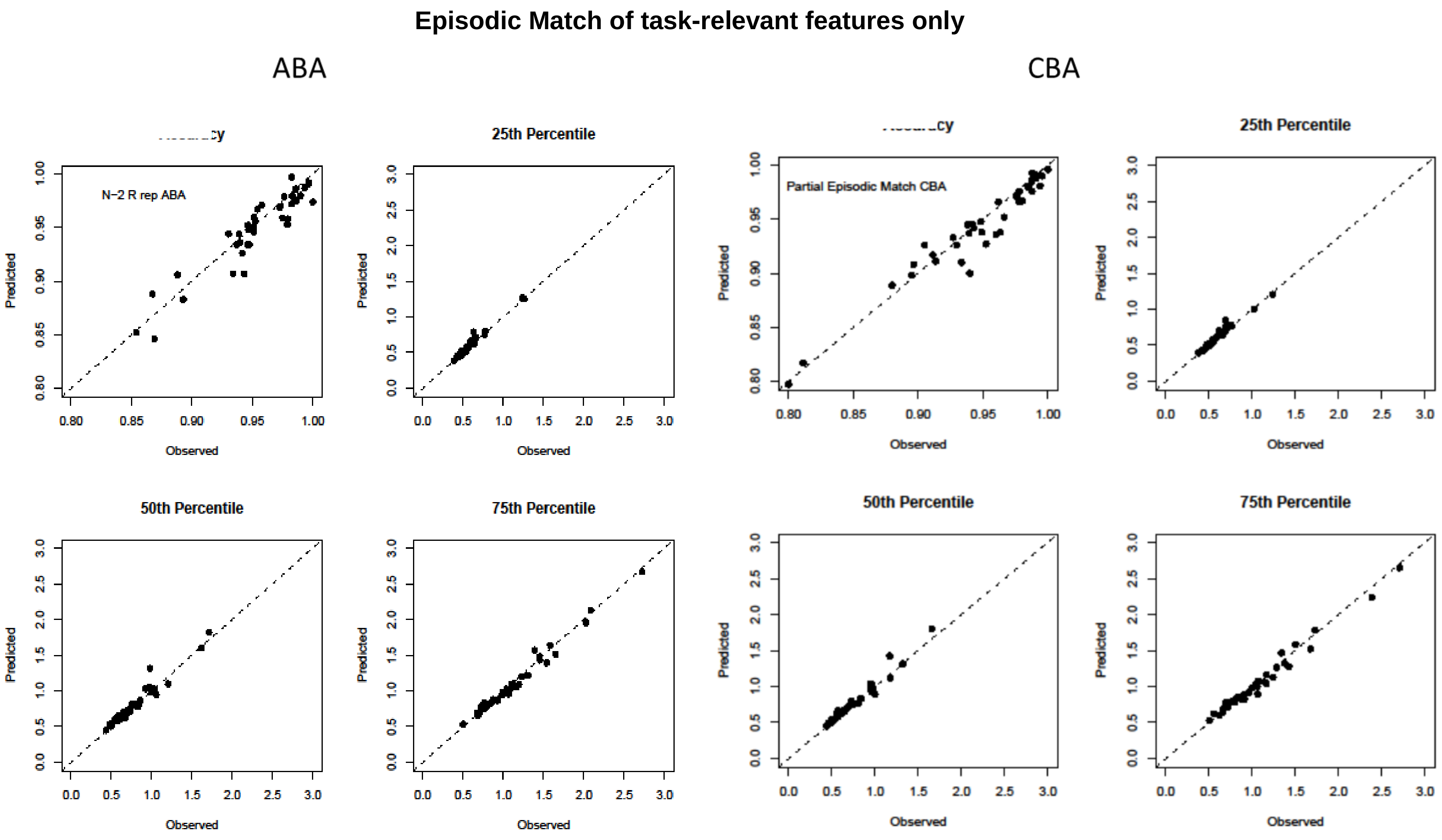

Figure S4 (continued).

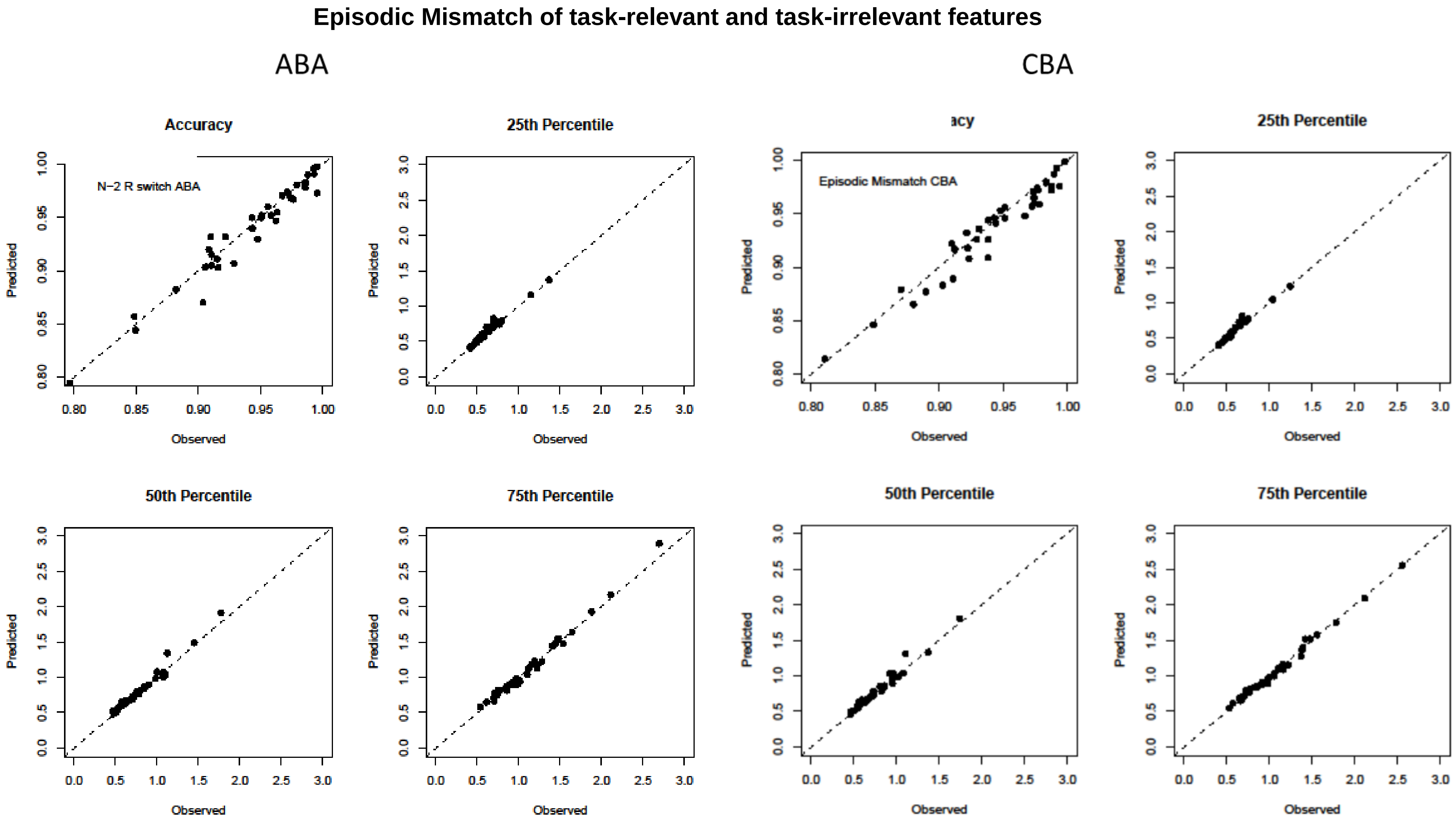

References

Grange, J. A. (2018). Does task activation in task switching influence inhibition or episodic interference? *Experimental Psychology, 65*, 393-404. https://doi.org/10.1027/1618-3169/a000423

Grange, J. A., & Juvina, I. (2015). The effect of practice on N–2 repetition costs in set switching. *Acta Psychologica, 154*, 14–25. https://doi.org/10.1016/j.actpsy.2014.11.003

Grange, J. A., Kedra, P., & Walker, A. (2019). The effect of practice on inhibition in task switching: Controlling for episodic retrieval. *Acta Psychologica, 192*, 59–72. https://doi.org/10.1016/j.actpsy.2018.10.006

Grange, J. A., Kowalczyk, A. W., & O’Loughlin, R. (2017). The effect of episodic retrieval on inhibition in task switching. *Journal of Experimental Psychology: Human Perception and Performance, 43*, 1568–1583. https://doi.org/10.1037/xhp0000411

Hedge, C., Vivian-Griffiths, S., Powell, G., Bompas, A., & Sumner, P. (2019). Slow and steady? Strategic adjustments in response caution are moderately reliable and correlate across tasks. *Consciousness and Cognition, 75*, Article 102797. https://doi.org/10.1016/j.concog.2019.102797

Kowalczyk, A. W., & Grange, J. A. (2019). The effect of episodic retrieval on inhibition in task switching: A diffusion model analysis. *Psychological Research, 84*(7), 1965–1999. https://doi.org/10.1007/s00426-019-01206-1

Lerche, V., Voss, A., & Nagler, M. (2017). How many trials are required for parameter estimation in diffusion modeling? A comparison of different optimization criteria. *Behavior Research Methods, 49*(2), 513–537. https://doi.org/10.3758/s13428-016-0740-2

Mayr, U. (2002). Inhibition of action rules. *Psychonomic Bulletin & Review, 9*, 93–99. https://doi.org/10.3758/BF031 96261 .

Moretti, L., Koch, I., Steinhauser, M., & Schuch, S. (2021). Errors in task switching: Investigating error aftereffects in a N-2 repetition cost paradigm. *Journal of Experimental Psychology: Learning, Memory, and Cognition*, *47*(10), 1720–1737. https://doi.org/10.1037/xlm0001034

Scheil, J. (2016). Effects of absolute and relative practice on N–2 repetition costs. *Acta Psychologica*, *164*, 65–69. https://doi.org/10.1016/j.actpsy.2015.12.011

Schmiedek, F., Oberauer, K., Wilhelm, O., Süss, H. M., & Wittmann, W. W. (2007). Individual differences in components of reaction time distributions and their relations to working memory and intelligence. *Journal of Experimental Psychology: General, 136*, 414–429. https://doi.org/10.1037/0096-3445.136.3.414

Schuch, S. (2016). Task inhibition and response inhibition in older versus younger adults: A diffusion model analysis. *Frontiers in Psychology, 7*, Article 1722. http://dx.doi.org/10.3389/fpsyg.2016.01722

Schuch, S. & Grange, J.A. (2019). Increased cognitive control after task conflict? Investigating the N-3 effect in task switching. *Psychological Research, 83*, 1703–1721. doi: 10.1007/s00426-018-1025-4

Schuch, S., & Konrad, K. (2017). Investigating task inhibition in children versus adults: A diffusion model analysis. *Journal of Experimental Child Psychology, 156*, 143–167. https://doi.org/10.1016/j.jecp.2016.11.012.

Schuch, S., & Dignath, D. (2020). Task conflict biases decision making. *Journal of Experimental Psychology: General. 150*(5), 864–872. https://doi.org/10.1037/xge0000908

Voss, A., & Voss, J. (2007). Fast-dm: A free program for efficient diffusion model analysis. *Behavior Research Methods, 39*(4), 767–775. https://doi.org/10.3758/BF03192967

Voss, A., Voss, J., & Lerche, V. (2015). Assessing cognitive processes with diffusion model analyses: A tutorial based on fast-dm-30. *Frontiers in Psychology, 6*, 336.
